# Investigating the causal mediating effect of type 2 diabetes on the relationship between traits and systolic blood pressure: A two-step Mendelian randomization study

**DOI:** 10.3389/fendo.2022.1090867

**Published:** 2022-12-16

**Authors:** Xuezeng Hao, Weixin Li, Ruiqing Shi, Qiuhong Wang

**Affiliations:** ^1^ Dongzhimen Hospital of Beijing University of Chinese Medicine, Beijing, China; ^2^ Institute of Acupuncture and Moxibustion, China Academy of Chinese Medical Sciences, Beijing, China; ^3^ Respiratory Endocrine Department, Beijing Fengtai You′anmen Hospital, Beijing, China; ^4^ Department of Endocrinology, Guang’anmen Hospital, China Academy of Chinese Medical Sciences, Beijing, China

**Keywords:** type 2 diabetes, blood pressure, hypertension, mediating effect, Mendelian randomization

## Abstract

**Background:**

Type 2 diabetes mellitus (T2DM) and hypertension commonly coexist, and we presumed that T2DM might mediate the relationship between some shared risk factors and systolic blood pressure (SBP).

**Methods:**

The causal association between T2DM and SBP was first confirmed using Mendelian randomization (MR) analyses, and a two-step MR design was then used to test the causal mediating effect of T2DM on the relationship between 107 traits and SBP using summary statistics from genome-wide association studies.

**Results:**

T2DM was causally associated with SBP. The univariable MR of the two-step causal mediation analyses suggested that 44 and 45 of the 107 traits had causal associations with T2DM and SBP, respectively. Five of the 27 traits that were significantly associated with both T2DM and SBP could not be reversely altered by T2DM and were included in the second step of the causal mediation analyses. The results indicated that most of the investigated traits causally altered SBP independent of T2DM, but the partial causal mediating effect of T2DM on the association between fasting insulin and SBP was successfully identified with a mediation proportion of 33.6%.

**Conclusions:**

Our study provides novel insights into the role of risk factors in the comorbidity of T2DM and high blood pressure, which is important for long-term disease prevention and management.

## Introduction

Type 2 diabetes mellitus (T2DM) and hypertension, commonly found to coexist (1–3), share many risk factors, including physical inactivity, alcohol consumption, and being overweight (4, 5). Additionally, an elevated risk of arterial stiffness, which contributes to the development of hypertension, is correlated with hyperglycemia and T2DM (6–8). Thus, we presumed that T2DM might mediate the relationship between shared risk factors and blood pressure. Determining these effects is important for understanding the underlying mechanisms of the comorbidities of T2DM and hypertension, as well as for long-term disease prevention and management.

Mendelian randomization (MR), a method implementing genetic instruments as a proxy for exposures, is a potent technique used for inferring the causality of exposures and outcomes free from bias due to residual confounding and reverse causality based on three core assumptions (9). Two-step MR is a novel strategy based on the well-established MR framework to improve causal inference for mediation analysis. The causal effect of exposure on outcome independent of (direct effect) or *via* (indirect effect) a mediator can be estimated in causal mediation analyses based on two-step MR (10, 11). No individual-level data are required by two-step MR analyses because they use genome-wide association studies (GWAS) summary statistics of traits and phenotypes, which are normally generated using populations with large sample sizes (12). Additionally, the accessibility to GWAS datasets facilitates the investigation of the mediating effect of T2DM on the association between many traits and blood pressure.

MR studies have been conducted to investigate the causal effect of risk factors on T2DM (13–15) and blood pressure alteration (16, 17). In addition, a two-step MR design has been successfully used to distinguish the direct effects of risk factors on atherosclerotic cardiovascular disease from those mediated by T2DM (18). However, to our knowledge, the potential causal mediating effect of T2DM on the relationship between risk factors and blood pressure is yet to be explored. Therefore, in the present study, we examined the causal association of T2DM with systolic blood pressure (SBP) and then performed causal mediation analyses based on two-step MR to systematically assess the potential mediating effect of T2DM on the causal association of risk factors with SBP.

## Methods

### Study design

A two-step MR design was used to test the causal mediation effect of T2DM (mediator) on the relationship between the traits (exposure) and SBP (outcome). First, the causal associations of 107 traits (**Supplementary Figure 1**) with T2DM and SBP (total effect) were studied using univariable MR as the first step of the two-step MR analyses. In addition, reverse univariable MR was conducted to examine whether these traits could be caused by T2DM because a reciprocal association between exposure and mediator was not allowed in the mediation analyses. Thus, only the traits that had a causal association with T2DM and SBP, but were not causally changed by T2DM, were included in the second step of the two-step MR analyses. Next, the direct effect of traits on SBP was calculated using multivariable MR, in which T2DM was set as the covariable. The indirect effects of traits on SBP were estimated by multiplying the beta coefficient from the causal association of traits with T2DM by those from the causal effect of T2DM on SBP with the adjustment of the trait as a covariable. In sum, the total, direct, and indirect effects in the causal mediation analyses were estimated using a two-step MR.

### Data sources

The trait selection procedure (**Supplementary Figure 1**) was similar to that used in a recent publication (18), in which GWAS summary statistics datasets from European/mixed ancestry, both sexes, and the largest population in the IEU OpenGWAS database were used. Most GWAS summary statistics of exposure traits were from the United Kingdom Biobank (UKBB). For the mediator and outcome data, GWAS datasets of T2DM and SBP were obtained from the Diabetes Meta-analysis of Trans-ethnic Association Studies (DIAMANTE) Consortium (19) and International Consortium of Blood Pressure (20), respectively. Detailed information is provided in **Supplementary Table 1**.

### Statistical methods

Instrumental variables (IVs) for exposure traits were selected according to several criteria in the univariable MR analyses. First, IVs should be strongly associated with exposure traits (*P* < 5×10^−8^). Second, IVs should be independent of each other, as quantified by linkage disequilibrium (LD) of R^2^ < 0.001, which was achieved by clumping with a 10 Mb window. Third, the IVs for each trait should have at least 10 variants, and the single nucleotide polymorphisms (SNPs) should be biallelic. The inverse-variance weighted (IVW) method, weighted median method, and MR-Egger were used in the univariable MR analyses, in which the IVW method was considered the main method because of its high statistical power when the selected IVs were valid (21). The MR-Egger intercept test was used to examine potential horizontal pleiotropy, and instrument strength was estimated using conditional F-statistics. Multiple comparisons were corrected using a 5% false-discovery rate (FDR). The code for two-step MR analyses was adapted from a published work (18), in which univariable and multivariable MR analyses were performed using the TwoSampleMR package and MVMR package in R, respectively.

## Results

**Figure 1:**
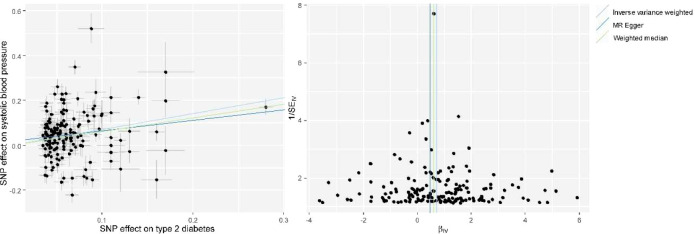
Scatter plot (*left*) and funnel plot (*right*) of Mendelian randomization (MR) analyses showing that T2DM is causally associated with increased systolic blood pressure (SBP). MR, Mendelian Randomization; SNP, single nucleotide polymorphism; IV, instrumental variable; T2DM, type 2 diabetes mellitus; SBP, systolic blood pressure.

We used univariable MR to test the causal association between T2DM and SBP, and the results from MR analysis using the IVW method revealed a significant association of genetically predicted T2DM with SBP (beta, 95% confidence intervals [CIs] and *P* were 0.71, 0.49–0.93 and 1.80×10^-10^, respectively) (**Figure 1**, **Supplementary Table 2**). MR sensitivity analyses with weighted median and MR-Egger methods indicated the same direction of association as the IVW method (**Figure 1**, **Supplementary Table 2**). Leave-one-out sensitivity analysis showed that the causal effect of T2DM on SBP was not driven by a specific SNP (**Supplementary Figure 2**). The evaluation of horizontal pleiotropy using the intercept term of the MR-Egger method suggested that horizontal pleiotropy was not significant (*P* = 0.30) in the analyses. MR-PRESSO also revealed a consistent causal association between T2DM and SBP after excluding potential outlier IVs (*P* = 1.38×10^-17^).

**Figure 2:**
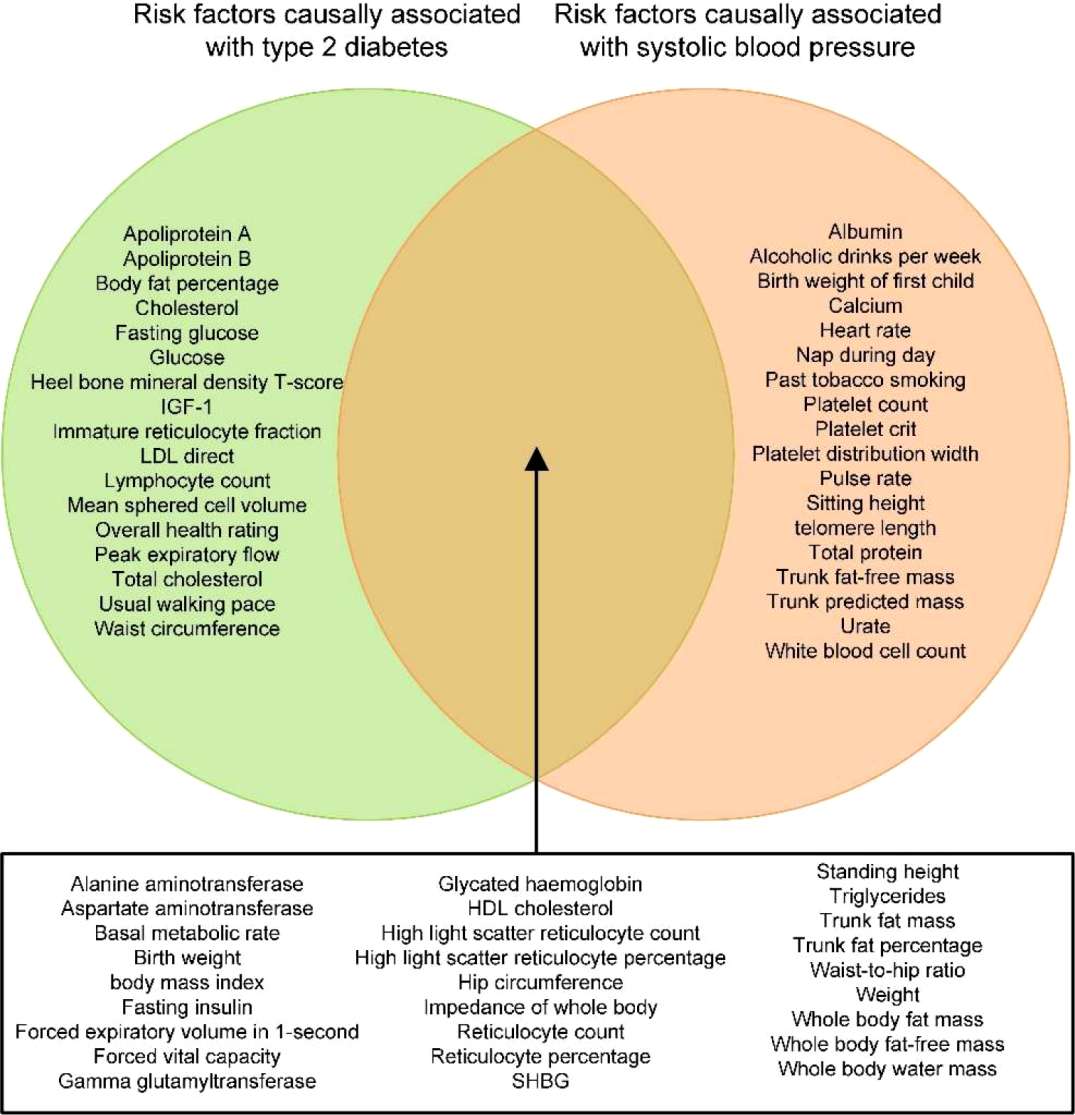
Venn diagram depicting the traits that are causally associated with type 2 diabetes mellitus (T2DM) and/or SBP. IGF-1, insulin-like growth factor-1; HDL, high-density lipoprotein; LDL, low-density lipoprotein; SHBG, sex hormone binding globulin; T2DM, type 2 diabetes mellitus; SBP, systolic blood pressure.

**Figure 3:**
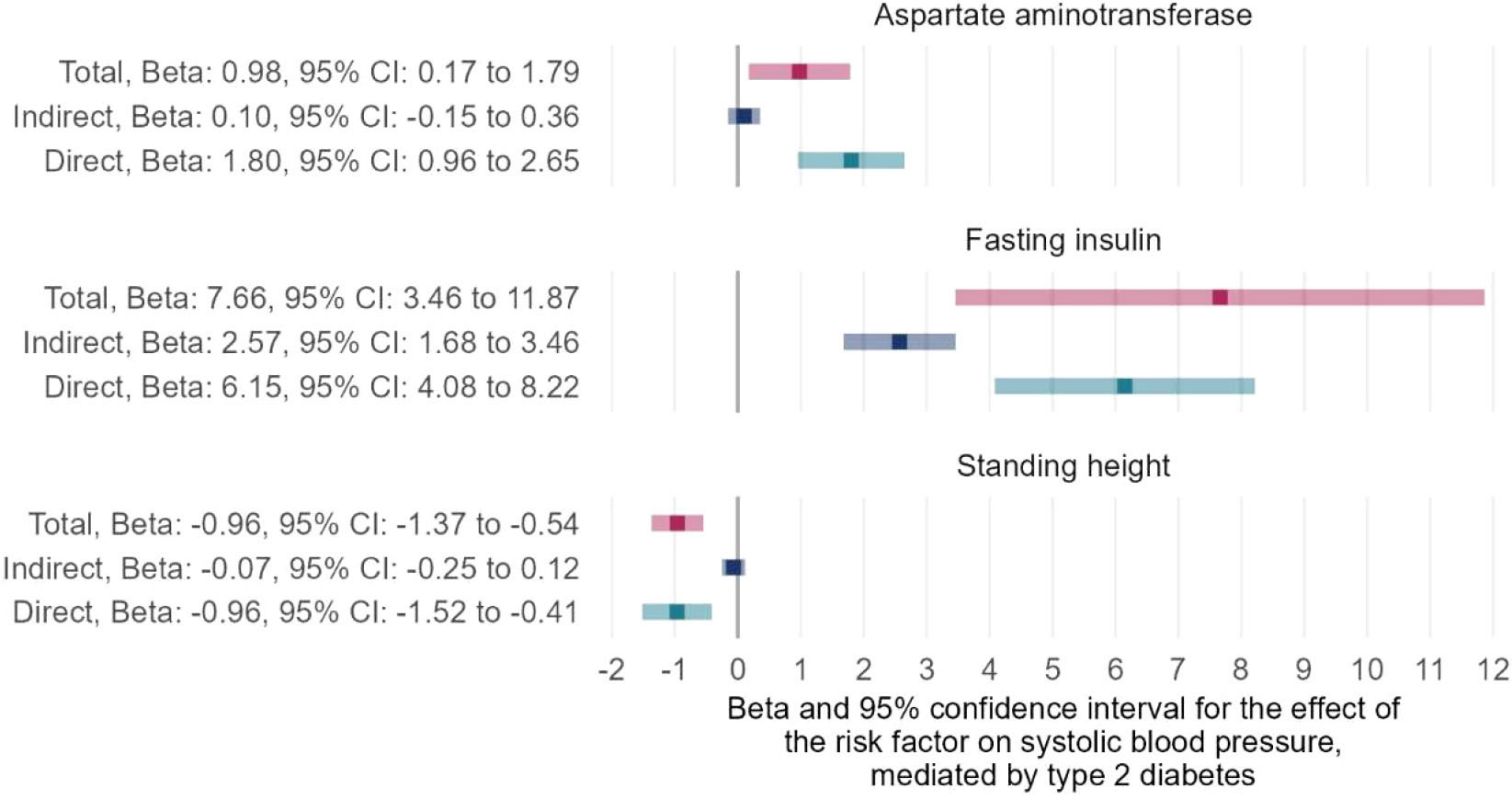
Two-step MR for mediation analyses showing the total, indirect (mediated by T2DM), and direct (independent of T2DM) effects of the traits on SBP. MR, Mendelian Randomization; T2DM, type 2 diabetes mellitus; SBP, systolic blood pressure.

After performing the trait exclusion procedure according to the criteria listed in the flowchart (**Supplementary Figure 1**), a final set of 107 traits from the IEU open GWAS database was included in the current study. Additional information on these traits, as well as their respective GWAS summary datasets, is included in **Supplementary Table 1**. The univariable MR of the two-step causal mediation analyses suggested that 44 and 45 of the 107 traits had causal associations with T2DM and SBP, respectively (**Supplementary Tables 3–5**). In addition, the Venn diagram indicates that 27 traits were significantly associated with both T2DM and SBP (**Figure 2**). Bidirectional univariable MR revealed that 22 of these traits could be reversely caused by T2DM and thus were excluded in the second step of the causal mediation analyses, which is presented in **Supplementary Tables 5**. For the included five traits (i.e., fasting insulin, trunk fat percentage, hip circumference, standing height, aspartate aminotransferase), multivariable MR analyses showed similar direct and total effects, indicating that adjustment of T2DM as a covariable did not alter the significance of the association between these traits and SBP (**Supplementary Tables 6, 7**). Furthermore, the direction of the indirect effects of three traits favored a potential mediating effect of T2DM on the causal association between traits and SBP (**Supplementary Table 7**, **Figure 3**), and the proportion of the medicating effect by T2DM for the traits “fasting insulin,” “aspartate aminotransferase,” and “standing height” was 33.6%, 10.2%, and 6.9%, respectively (**Supplementary Table 7**). The conditional F-statistics of the investigated traits in the multivariable MR ranged from 9.2 to 43.0, representing good instrument strength. Thus, the causal medication analyses using a two-step MR design showed that most investigated traits causally altered SBP independent of T2DM, but the partial causal mediating effect of T2DM on the association between fasting insulin and SBP was successfully identified.

## Discussion

In this study, univariable MR analyses indicated a causal relationship between T2DM and SBP, as well as the causal effects of numerous traits on T2DM and SBP. Many common risk factors for the two outcomes of interest were identified, including glycemic traits (e.g., fasting insulin and glycated hemoglobin), blood lipid indices (e.g., high-density lipoprotein [HDL] and triglycerides), anthropometric markers (e.g., body mass index, standing height, waist-to-hip ratio, and whole body fat mass), and pulmonary function indicators (e.g., forced vital capacity and forced expiratory volume in 1-second). However, most of these common risk factors (22 of 27 risk factors) of T2DM and SBP were not included in the second step of the two-step MR analyses because causal mediation analysis required no reciprocal causal association of the mediator (T2DM) with exposure (common risk factors). Two-step MR for mediation analyses suggested that three of the included five traits had indirect effects with a direction favoring a potential mediating effect of T2DM, and the causal association between fasting insulin and SBP could be partially mediated by T2DM with an estimated mediation proportion of 33.57%.

Diabetes and hypertension frequently occur together (22–24). For instance, patients with diabetes are twice as likely to have hypertension than that of non-diabetic individuals (25). Furthermore, a recent MR analysis revealed a causal relationship between T2DM and hypertension, in which a higher SBP, but not diastolic blood pressure (DBP), can be induced by T2DM (26). SBP refers to the peak blood pressure recorded during heart contraction, while DBP refers to the blood pressure recorded when the heart rests between beats. Since the Framingham study in 1980 showed that systolic hypertension is a more powerful indicator of cardiovascular events than diastolic hypertension (27), SBP has been of great importance (28–30). Moreover, the hypertension management guidelines by the American College of Cardiology (ACC) and American Heart Association (AHA) removed DBP from the assessment of cardiovascular risk in 2017 (31). Thus, we selected SBP as the outcome in the current study, and our findings provide consistent evidence supporting the causal effect of T2DM on SBP. Mechanistically, the natural course of diabetes promotes the development of high blood pressure. For example, hyperglycemia and hyperinsulinemia lead to peripheral artery resistance by vascular remodeling and narrowing and increase circulatory blood volume by sodium reabsorption and hyperosmolarity, which eventually elevates heart contractility and blood pressure (32, 33).

Diabetes and hypertension share a considerable number of common pathophysiological pathways as end results of the metabolic syndrome (22, 24, 34). These pathways involve multiple key players, such as the renin-angiotensin-aldosterone system (RAAS) (35), obesity (36, 37), inflammation (38, 39), oxidative stress (40) and insulin resistance (41), which interact with each other and form a vicious cycle. For instance, RAAS activity is inappropriately upregulated in obese individuals (42), which may induce insulin resistance through the regulation of Ang II type 1 receptor, resulting in increased oxidative stress in adipocytes, skeletal muscle, and cardiovascular tissue, aggravating the development of diabetes and hypertension (34, 35). Thus, diabetes and hypertension are expected to be associated with several common risk factors. Epidemiological data indicate that the waist-hip ratio and hip circumference are both closely associated with the risk of T2DM and blood pressure (43–46). MR studies further verified the clinical observation of the potent contributions of the waist-hip ratio and body mass index on diabetes or cardiovascular risk (e.g., high blood pressure) from a genetic perspective (47, 48). In addition, a review pointed out that unhealthy fat distribution in the body increases cardiometabolic risk, whereas a high amount of fat in the lower part of the body may play a protective role against T2DM and cardiovascular diseases (49). One plausible explanation is that visceral abdominal obesity, measured by waist-hip circumference, is detrimental to metabolic activity and the cardiovascular system (50). Another explanation is that subcutaneous adipose tissue in the lower part of the body has a less negative impact on metabolism than that in the viscera in the upper part of the body (51–53). In accordance with previous studies, we found evidence for several anthropometric markers as common risk factors that could lead to both T2DM and SBP, such as body mass index, hip circumference, waist-to-hip ratio, and whole-body fat mass. As for other traits, the association between lung function and diabetes (54) and blood pressure (55, 56), as well as the association of glycemic traits and lipids with blood pressure (57, 58), have been epidemiologically and clinically well-accepted. In the present study, we illustrated the causal relationship of 27 common risk factors with both T2DM and SBP, including anthropometric markers, pulmonary function indicators, glycemic traits, and blood lipid indices.

Insulin is a hormone that can significantly affect blood glucose levels, and the abnormal regulation of insulin contributes to the pathogenesis of diabetes. Fasting insulin is considered part of the clinical definition of T2DM and is an effective clinical tool for predicting prediabetes (59). In addition, insulin plays an important role in regulating SBP independently of diabetes, with evidence that insulin is correlated with SBP in non-diabetic individuals (60, 61). Moreover, an animal study demonstrated that excessive insulin increased heart function and significantly pushed up SBP (62). Biologically, insulin increases the activity of Na^+^/K^+^-ATPase to promote the transport of sodium ions into the blood vessels through renal tubule cells (63). Thus, insulin resistance with compensatory hyperinsulinemia facilitates sodium retention to elevate blood pressure, independent of diabetes (64). Along with the progression of diabetes induced by insulin deficiency, vascular fibrosis and stiffness and activated RAAS drive up blood pressure (33, 65, 66), which reflects the indirect effect of insulin on SBP *via* diabetes. Our results align with these mechanisms, indicating that the modulating effect of fasting insulin on SBP could be independent of (direct effects) or *via* (indirect effects) diabetes. In addition, considering a broad range of risk factors for blood pressure control in individuals with diabetes is necessary, given our results that most traits influence SBP independent of diabetes.

Two-step MR can infer causality when analyzing the mediating effect of T2DM on the association between various traits and SBP, which is a major strength of the current study because the biases caused by residual confounding and reverse causality are diminished by the MR design. In addition, the use of summary statistics from large GWAS for exposures, mediators, and outcomes increases the power of the statistical analyses. Moreover, multiple MR methods, including the weighted median method, MR-Egger, and multivariable MR, were used for sensitivity analyses, by which horizontal pleiotropy and instrument strength were estimated. However, the present study has several limitations. First, the relatively large number of traits included in this study increased the burden of multiple comparison correction, thereby altering the number of traits that could be passed on to the second step of the two-step MR analysis. Second, the potential mediating effect might have been underestimated because of the higher statistical power required for multivariable MR analyses. Third, horizontal pleiotropy is ubiquitous in MR analyses, which may have introduced bias in the current study.

## Conclusion

T2DM causally increases SBP and partially mediates the causal association between fasting insulin and SBP. Other eligible traits included in the causal medication analyses altered SBP independent of T2DM. Our study provides novel insights into the role of risk factors in the comorbidity of T2DM and high blood pressure.

## Data availability statement

The original contributions presented in the study are included in the article/**Supplementary Material**. Further inquiries can be directed to the corresponding authors.

## Ethics statement

The GWAS summary statistics used in this study were approved by the relevant review board. Informed consent was also obtained from all participants.

## Author contributions

XH and WL designed the study. XH, WL and RS performed the statistical analyses and drafted the manuscript. QW critically reviewed the manuscript. All authors read and approved the final manuscript.
