# Supplementary material for: Investigating the causal mediating effect of type 2 diabetes on the relationship between traits and systolic blood pressure: A two-step Mendelian randomization study
Source: Front Endocrinol (Lausanne). 2022 Dec 16;13:1090867. doi: 10.3389/fendo.2022.1090867 (PMC9800519; doi:10.3389/fendo.2022.1090867)
Supplement: Supplementary file 1 [file DataSheet_1.docx]

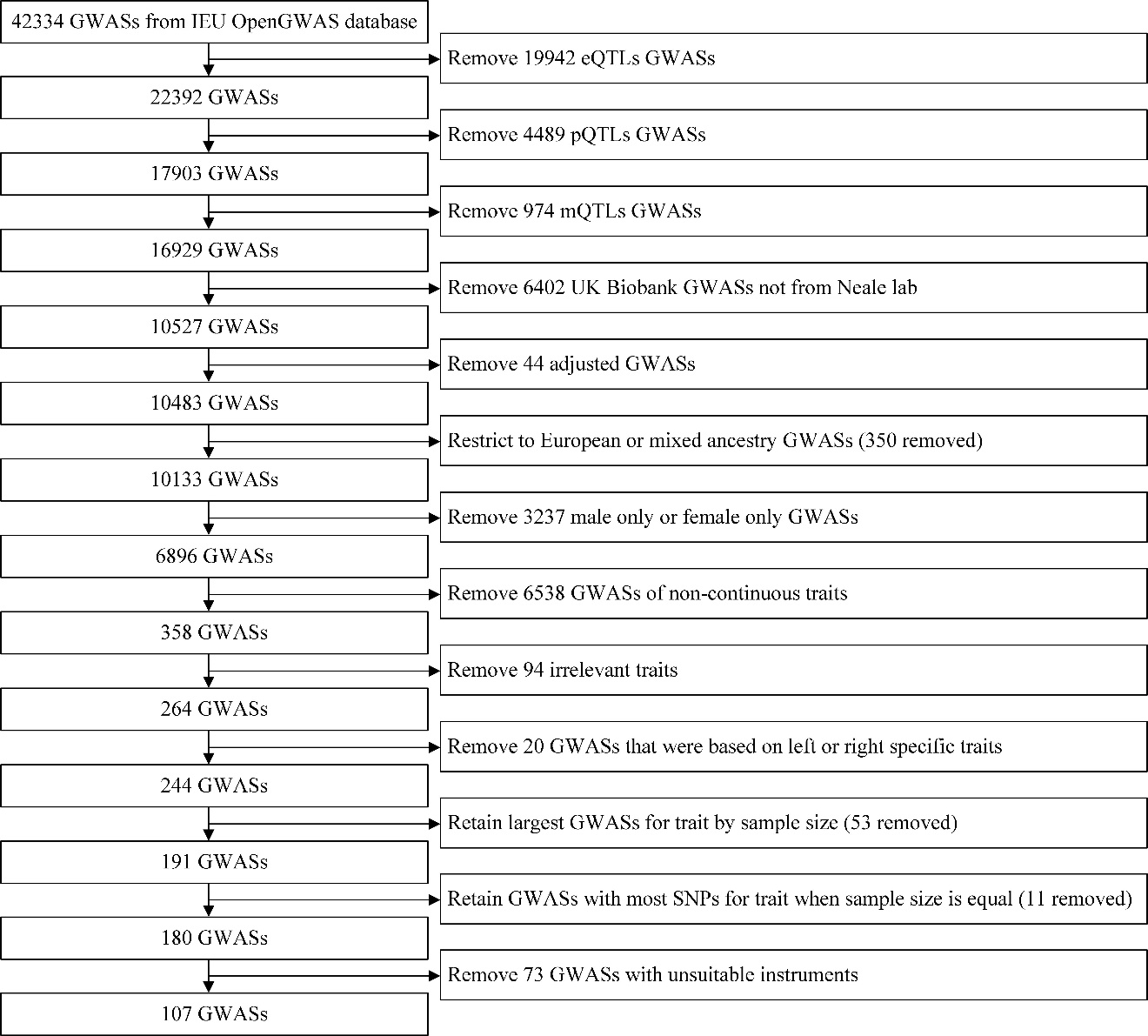


Supplementary Figure 1. Flowchart indicating the trait selection procedure in the causal mediation analyses


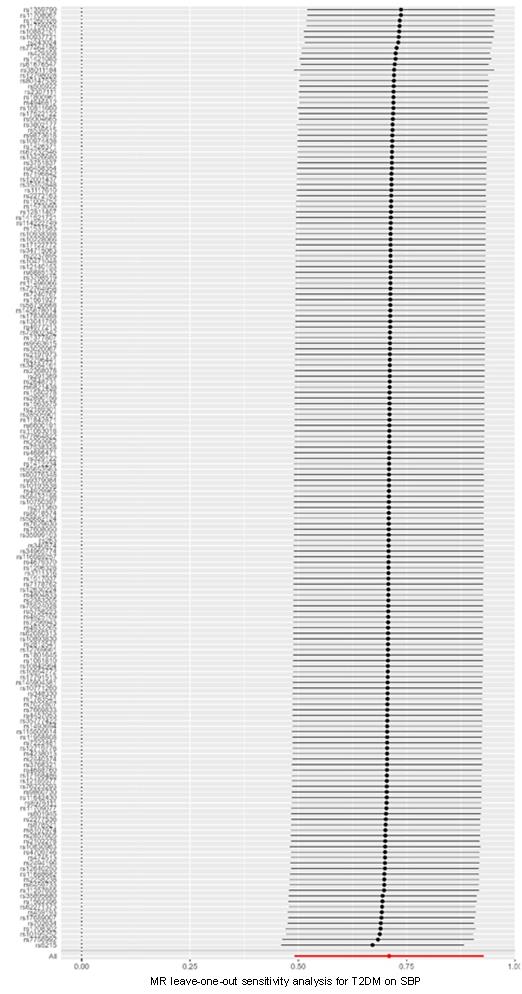


Supplementary Figure 2. Leave-one-out plot as MR sensitivity analysis for the causal effect of T2DM on SBP

T2DM, type 2 diabetes mellitus; SBP, systolic blood pressure.

Supplementary Table 1. GWAS summary datasets used in causal mediation analyses.

| trait | id | year | author | consortium | sex | population | sample_size | type |
| --- | --- | --- | --- | --- | --- | --- | --- | --- |
| Sleep duration | ukb-a-9 | 2017 | Neale | Neale Lab | Males and Females | European | 335410 | risk_factor |
| Lymphocyte percentage | ukb-d-30180_irnt | 2018 | Neale lab | UK Biobank | Males and Females | European | 349861 | risk_factor |
| Basal metabolic rate | ukb-a-268 | 2017 | Neale | Neale Lab | Males and Females | European | 331307 | risk_factor |
| Pulse rate automated reading | ukb-a-3 | 2017 | Neale | Neale Lab | Males and Females | European | 317756 | risk_factor |
| Forced vital capacity (FVC) | ukb-a-336 | 2017 | Neale | Neale Lab | Males and Females | European | 307638 | risk_factor |
| Body fat percentage | ukb-a-264 | 2017 | Neale | Neale Lab | Males and Females | European | 331117 | risk_factor |
| Mean corpuscular volume | ukb-d-30040_irnt | 2018 | Neale lab | UK Biobank | Males and Females | European | 350473 | risk_factor |
| Past tobacco smoking | ukb-a-17 | 2017 | Neale | Neale Lab | Males and Females | European | 310749 | risk_factor |
| Urea | ukb-d-30670_irnt | 2018 | Neale lab | UK Biobank | Males and Females | European | 361194 | risk_factor |
| Sleeplessness / insomnia | ukb-a-13 | 2017 | Neale | Neale Lab | Males and Females | European | 336965 | risk_factor |
| Creatinine (enzymatic) in urine | ukb-a-333 | 2017 | Neale | Neale Lab | Males and Females | European | 327525 | risk_factor |
| Standing height | ukb-a-389 | 2017 | Neale | Neale Lab | Males and Females | European | 336474 | risk_factor |
| High light scatter reticulocyte percentage | ukb-d-30290_irnt | 2018 | Neale lab | UK Biobank | Males and Females | European | 344729 | risk_factor |
| Total protein | ukb-d-30860_irnt | 2018 | Neale lab | UK Biobank | Males and Females | European | 361194 | risk_factor |
| HDL cholesterol | ukb-d-30760_irnt | 2018 | Neale lab | UK Biobank | Males and Females | European | 361194 | risk_factor |
| Red blood cell (erythrocyte) count | ukb-d-30010_irnt | 2018 | Neale lab | UK Biobank | Males and Females | European | 350475 | risk_factor |
| telomere length | ieu-b-4879 | 2021 | Codd |  | Males and Females | European | 472174 | risk_factor |
| Whole body fat-free mass | ukb-a-266 | 2017 | Neale | Neale Lab | Males and Females | European | 331291 | risk_factor |
| Heart rate | ieu-a-1056 | 2013 | den Hoed M | HRgene consortium | Males and Females | Mixed | 92355 | risk_factor |
| Drive faster than motorway speed limit | ukb-a-8 | 2017 | Neale | Neale Lab | Males and Females | European | 306030 | risk_factor |
| Albumin | ukb-d-30600_irnt | 2018 | Neale lab | UK Biobank | Males and Females | European | 361194 | risk_factor |
| Calcium | ukb-d-30680_irnt | 2018 | Neale lab | UK Biobank | Males and Females | European | 361194 | risk_factor |
| Mean platelet (thrombocyte) volume | ukb-d-30100_irnt | 2018 | Neale lab | UK Biobank | Males and Females | European | 350470 | risk_factor |
| Birth weight of first child | ukb-a-318 | 2017 | Neale | Neale Lab | Males and Females | European | 145558 | risk_factor |
| Triglycerides | ukb-d-30870_irnt | 2018 | Neale lab | UK Biobank | Males and Females | European | 361194 | risk_factor |
| Fasting glucose | ieu-b-114 | 2012 | Scott | MAGIC | Males and Females | European | 133010 | risk_factor |
| Haematocrit percentage | ukb-d-30030_irnt | 2018 | Neale lab | UK Biobank | Males and Females | European | 350475 | risk_factor |
| Alanine aminotransferase | ukb-d-30620_irnt | 2018 | Neale lab | UK Biobank | Males and Females | European | 361194 | risk_factor |
| Reticulocyte percentage | ukb-d-30240_irnt | 2018 | Neale lab | UK Biobank | Males and Females | European | 344728 | risk_factor |
| Gamma glutamyltransferase | ukb-d-30730_irnt | 2018 | Neale lab | UK Biobank | Males and Females | European | 361194 | risk_factor |
| Phosphate | ukb-d-30810_irnt | 2018 | Neale lab | UK Biobank | Males and Females | European | 361194 | risk_factor |
| Trunk fat percentage | ukb-a-290 | 2017 | Neale | Neale Lab | Males and Females | European | 331113 | risk_factor |
| Total bilirubin | ukb-d-30840_irnt | 2018 | Neale lab | UK Biobank | Males and Females | European | 361194 | risk_factor |
| Peak expiratory flow (PEF) | ukb-a-338 | 2017 | Neale | Neale Lab | Males and Females | European | 307638 | risk_factor |
| Reticulocyte count | ukb-d-30250_irnt | 2018 | Neale lab | UK Biobank | Males and Females | European | 344729 | risk_factor |
| Red blood cell (erythrocyte) distribution width | ukb-d-30070_irnt | 2018 | Neale lab | UK Biobank | Males and Females | European | 350473 | risk_factor |
| Heel bone mineral density (BMD) T-score automated | ukb-a-500 | 2017 | Neale | Neale Lab | Males and Females | European | 194398 | risk_factor |
| Mean corpuscular haemoglobin | ukb-d-30050_irnt | 2018 | Neale lab | UK Biobank | Males and Females | European | 350472 | risk_factor |
| Platelet count | ukb-d-30080_irnt | 2018 | Neale lab | UK Biobank | Males and Females | European | 350474 | risk_factor |
| Cystatin C | ukb-d-30720_irnt | 2018 | Neale lab | UK Biobank | Males and Females | European | 361194 | risk_factor |
| Weight | ukb-a-249 | 2017 | Neale | Neale Lab | Males and Females | European | 336227 | risk_factor |
| Vitamin D | ukb-d-30890_irnt | 2018 | Neale lab | UK Biobank | Males and Females | European | 361194 | risk_factor |
| Nap during day | ukb-a-12 | 2017 | Neale | Neale Lab | Males and Females | European | 337074 | risk_factor |
| High light scatter reticulocyte count | ukb-d-30300_irnt | 2018 | Neale lab | UK Biobank | Males and Females | European | 344729 | risk_factor |
| Urate | ukb-d-30880_irnt | 2018 | Neale lab | UK Biobank | Males and Females | European | 361194 | risk_factor |
| Adiponectin | ieu-a-1 | 2012 | Dastani Z | ADIPOGen | Males and Females | Mixed | 39883 | risk_factor |
| Testosterone | ukb-d-30850_irnt | 2018 | Neale lab | UK Biobank | Males and Females | European | 361194 | risk_factor |
| Cholesterol | ukb-d-30690_irnt | 2018 | Neale lab | UK Biobank | Males and Females | European | 361194 | risk_factor |
| Sodium in urine | ukb-a-335 | 2017 | Neale | Neale Lab | Males and Females | European | 326831 | risk_factor |
| Lymphocyte count | ukb-d-30120_irnt | 2018 | Neale lab | UK Biobank | Males and Females | European | 349856 | risk_factor |
| Trunk predicted mass | ukb-a-293 | 2017 | Neale | Neale Lab | Males and Females | European | 330995 | risk_factor |
| Platelet distribution width | ukb-d-30110_irnt | 2018 | Neale lab | UK Biobank | Males and Females | European | 350470 | risk_factor |
| Haemoglobin concentration | ukb-d-30020_irnt | 2018 | Neale lab | UK Biobank | Males and Females | European | 350474 | risk_factor |
| Waist-to-hip ratio | ieu-a-72 | 2015 | Shungin D | GIANT | Males and Females | Mixed | 224459 | risk_factor |
| Neutrophill count | ukb-d-30140_irnt | 2018 | Neale lab | UK Biobank | Males and Females | European | 349856 | risk_factor |
| Whole body water mass | ukb-a-267 | 2017 | Neale | Neale Lab | Males and Females | European | 331315 | risk_factor |
| Apoliprotein A | ukb-d-30630_irnt | 2018 | Neale lab | UK Biobank | Males and Females | European | 361194 | risk_factor |
| Neuroticism score | ukb-a-230 | 2017 | Neale | Neale Lab | Males and Females | European | 274108 | risk_factor |
| Morning/evening person (chronotype) | ukb-a-11 | 2017 | Neale | Neale Lab | Males and Females | European | 301143 | risk_factor |
| Urinary sodium-potassium ratio | ieu-b-72 | 2020 | Zanetti |  | Males and Females | European | 326938 | risk_factor |
| Mean reticulocyte volume | ukb-d-30260_irnt | 2018 | Neale lab | UK Biobank | Males and Females | European | 344728 | risk_factor |
| Hip circumference | ukb-a-388 | 2017 | Neale | Neale Lab | Males and Females | European | 336601 | risk_factor |
| White blood cell (leukocyte) count | ukb-d-30000_irnt | 2018 | Neale lab | UK Biobank | Males and Females | European | 350470 | risk_factor |
| Overall health rating | ukb-a-251 | 2017 | Neale | Neale Lab | Males and Females | European | 336020 | risk_factor |
| Neutrophill percentage | ukb-d-30200_irnt | 2018 | Neale lab | UK Biobank | Males and Females | European | 349861 | risk_factor |
| Mean sphered cell volume | ukb-d-30270_irnt | 2018 | Neale lab | UK Biobank | Males and Females | European | 344729 | risk_factor |
| SHBG | ukb-d-30830_irnt | 2018 | Neale lab | UK Biobank | Males and Females | European | 361194 | risk_factor |
| LDL direct | ukb-d-30780_irnt | 2018 | Neale lab | UK Biobank | Males and Females | European | 361194 | risk_factor |
| Immature reticulocyte fraction | ukb-d-30280_irnt | 2018 | Neale lab | UK Biobank | Males and Females | European | 344728 | risk_factor |
| Monocyte percentage | ukb-d-30190_irnt | 2018 | Neale lab | UK Biobank | Males and Females | European | 349861 | risk_factor |
| Aspartate aminotransferase | ukb-d-30650_irnt | 2018 | Neale lab | UK Biobank | Males and Females | European | 361194 | risk_factor |
| Creatinine | ukb-d-30700_irnt | 2018 | Neale lab | UK Biobank | Males and Females | European | 361194 | risk_factor |
| Lipoprotein A | ukb-d-30790_irnt | 2018 | Neale lab | UK Biobank | Males and Females | European | 361194 | risk_factor |
| Apoliprotein B | ukb-d-30640_irnt | 2018 | Neale lab | UK Biobank | Males and Females | European | 361194 | risk_factor |
| Neuroticism | ieu-a-1007 | 2016 | Okbay | SSGAC | Males and Females | European | 170911 | risk_factor |
| Eosinophill percentage | ukb-d-30210_irnt | 2018 | Neale lab | UK Biobank | Males and Females | European | 349861 | risk_factor |
| Total cholesterol | ieu-a-301 | 2013 | Willer CJ | GLGC | Males and Females | Mixed | 187365 | risk_factor |
| Getting up in morning | ukb-a-10 | 2017 | Neale | Neale Lab | Males and Females | European | 336501 | risk_factor |
| Mean time to correctly identify matches | ukb-a-199 | 2017 | Neale | Neale Lab | Males and Females | European | 335139 | risk_factor |
| Whole body fat mass | ukb-a-265 | 2017 | Neale | Neale Lab | Males and Females | European | 330762 | risk_factor |
| Fasting insulin | ieu-b-116 | 2012 | Scott | MAGIC | Males and Females | European | 108557 | risk_factor |
| Direct bilirubin | ukb-d-30660_irnt | 2018 | Neale lab | UK Biobank | Males and Females | European | 361194 | risk_factor |
| Body fat | ieu-a-999 | 2016 | Lu |  | Males and Females | European | 100716 | risk_factor |
| Daytime dozing / sleeping (narcolepsy) | ukb-a-15 | 2017 | Neale | Neale Lab | Males and Females | European | 336082 | risk_factor |
| Alcohol intake frequency. | ukb-a-25 | 2017 | Neale | Neale Lab | Males and Females | European | 336965 | risk_factor |
| Alcoholic drinks per week | ieu-b-73 | 2019 | Liu, M | GWAS and Sequencing Consortium of Alcohol and Nicotine use | Males and Females | European | 335394 | risk_factor |
| Glycated haemoglobin | ukb-d-30750_irnt | 2018 | Neale lab | UK Biobank | Males and Females | European | 361194 | risk_factor |
| Mean corpuscular haemoglobin concentration | ukb-d-30060_irnt | 2018 | Neale lab | UK Biobank | Males and Females | European | 350468 | risk_factor |
| Trunk fat mass | ukb-a-291 | 2017 | Neale | Neale Lab | Males and Females | European | 331093 | risk_factor |
| Cigarettes per Day | ieu-b-25 | 2019 | Liu, M | GWAS and Sequencing Consortium of Alcohol and Nicotine use | Males and Females | European | 337334 | risk_factor |
| Impedance of whole body | ukb-a-269 | 2017 | Neale | Neale Lab | Males and Females | European | 331284 | risk_factor |
| Birth weight | ukb-a-198 | 2017 | Neale | Neale Lab | Males and Females | European | 193063 | risk_factor |
| Usual walking pace | ukb-a-513 | 2017 | Neale | Neale Lab | Males and Females | European | 335349 | risk_factor |
| IGF-1 | ukb-d-30770_irnt | 2018 | Neale lab | UK Biobank | Males and Females | European | 361194 | risk_factor |
| Fluid intelligence score | ukb-a-196 | 2017 | Neale | Neale Lab | Males and Females | European | 108818 | risk_factor |
| Basophill percentage | ukb-d-30220_irnt | 2018 | Neale lab | UK Biobank | Males and Females | European | 349861 | risk_factor |
| Waist circumference | ukb-a-382 | 2017 | Neale | Neale Lab | Males and Females | European | 336639 | risk_factor |
| Monocyte count | ukb-d-30130_irnt | 2018 | Neale lab | UK Biobank | Males and Females | European | 349856 | risk_factor |
| Current tobacco smoking | ukb-a-16 | 2017 | Neale | Neale Lab | Males and Females | European | 337030 | risk_factor |
| Alkaline phosphatase | ukb-d-30610_irnt | 2018 | Neale lab | UK Biobank | Males and Females | European | 361194 | risk_factor |
| Platelet crit | ukb-d-30090_irnt | 2018 | Neale lab | UK Biobank | Males and Females | European | 350471 | risk_factor |
| Forced expiratory volume in 1-second (FEV1) | ukb-a-337 | 2017 | Neale | Neale Lab | Males and Females | European | 307638 | risk_factor |
| Sitting height | ukb-a-195 | 2017 | Neale | Neale Lab | Males and Females | European | 336172 | risk_factor |
| C-reactive protein | ukb-d-30710_irnt | 2018 | Neale lab | UK Biobank | Males and Females | European | 361194 | risk_factor |
| Trunk fat-free mass | ukb-a-292 | 2017 | Neale | Neale Lab | Males and Females | European | 331030 | risk_factor |
| body mass index | ieu-b-40 | 2018 | Yengo, L | GIANT | Males and Females | European | 681275 | risk_factor |
| Glucose | ukb-d-30740_irnt | 2018 | Neale lab | UK Biobank | Males and Females | European | 361194 | risk_factor |
| Type 2 diabetes | t2d | 2018 | Mahajan | DIAMANTE | Males and Females | European | 898130 | outcome |
| Systolic blood pressure | sbp | 2018 | Evangelou, E | International Consortium of Blood Pressure | Males and Females | European | 757601 | outcome |

Supplementary Table 2. MR analysis results of the effect of type 2 diabetes on systolic blood pressure.

| exposure | outcome | method | nsnp | b | se | pval |
| --- | --- | --- | --- | --- | --- | --- |
| type 2 diabetes | systolic blood pressure | MR Egger | 172 | 0.468 | 0.258 | 7.20E-02 |
| type 2 diabetes | systolic blood pressure | Weighted median | 172 | 0.611 | 0.104 | 4.22E-09 |
| type 2 diabetes | systolic blood pressure | Inverse variance weighted | 172 | 0.710 | 0.111 | 1.80E-10 |

Supplementary Table 3. The results of univariable MR showing the effect of traits on systolic blood pressure.

| exposure | outcome | method | nsnp | b | se | pval |
| --- | --- | --- | --- | --- | --- | --- |
| Trunk fat percentage | systolic blood pressure | MR Egger | 205 | -4.335 | 1.598 | 7.25E-03 |
| Trunk fat percentage | systolic blood pressure | Weighted median | 205 | 0.090 | 0.294 | 7.58E-01 |
| Trunk fat percentage | systolic blood pressure | Inverse variance weighted | 205 | -1.082 | 0.451 | 1.64E-02 |
| Trunk fat percentage | systolic blood pressure | Simple mode | 205 | 1.456 | 0.900 | 1.08E-01 |
| Trunk fat percentage | systolic blood pressure | Weighted mode | 205 | 0.911 | 0.517 | 7.95E-02 |
| Total cholesterol | systolic blood pressure | MR Egger | 77 | 0.741 | 0.390 | 6.16E-02 |
| Total cholesterol | systolic blood pressure | Weighted median | 77 | 1.004 | 0.157 | 1.50E-10 |
| Total cholesterol | systolic blood pressure | Inverse variance weighted | 77 | 0.307 | 0.248 | 2.15E-01 |
| Total cholesterol | systolic blood pressure | Simple mode | 77 | 0.622 | 0.310 | 4.84E-02 |
| Total cholesterol | systolic blood pressure | Weighted mode | 77 | 0.848 | 0.133 | 1.18E-08 |
| Gamma glutamyltransferase | systolic blood pressure | MR Egger | 206 | -0.163 | 0.438 | 7.11E-01 |
| Gamma glutamyltransferase | systolic blood pressure | Weighted median | 206 | 0.376 | 0.195 | 5.32E-02 |
| Gamma glutamyltransferase | systolic blood pressure | Inverse variance weighted | 206 | 0.783 | 0.242 | 1.19E-03 |
| Gamma glutamyltransferase | systolic blood pressure | Simple mode | 206 | 1.542 | 0.513 | 2.97E-03 |
| Gamma glutamyltransferase | systolic blood pressure | Weighted mode | 206 | 0.171 | 0.210 | 4.18E-01 |
| Heel bone mineral density (BMD) T-score automated | systolic blood pressure | MR Egger | 220 | -0.403 | 0.392 | 3.04E-01 |
| Heel bone mineral density (BMD) T-score automated | systolic blood pressure | Weighted median | 220 | -0.257 | 0.153 | 9.26E-02 |
| Heel bone mineral density (BMD) T-score automated | systolic blood pressure | Inverse variance weighted | 220 | 0.088 | 0.204 | 6.67E-01 |
| Heel bone mineral density (BMD) T-score automated | systolic blood pressure | Simple mode | 220 | -0.039 | 0.310 | 9.00E-01 |
| Heel bone mineral density (BMD) T-score automated | systolic blood pressure | Weighted mode | 220 | -0.253 | 0.126 | 4.52E-02 |
| Glycated haemoglobin | systolic blood pressure | MR Egger | 220 | 0.280 | 0.327 | 3.93E-01 |
| Glycated haemoglobin | systolic blood pressure | Weighted median | 220 | 0.510 | 0.137 | 1.89E-04 |
| Glycated haemoglobin | systolic blood pressure | Inverse variance weighted | 220 | 0.836 | 0.206 | 5.05E-05 |
| Glycated haemoglobin | systolic blood pressure | Simple mode | 220 | 0.833 | 0.378 | 2.87E-02 |
| Glycated haemoglobin | systolic blood pressure | Weighted mode | 220 | 0.745 | 0.123 | 5.20E-09 |
| Whole body fat-free mass | systolic blood pressure | MR Egger | 368 | -3.462 | 0.901 | 1.43E-04 |
| Whole body fat-free mass | systolic blood pressure | Weighted median | 368 | -2.132 | 0.273 | 5.26E-15 |
| Whole body fat-free mass | systolic blood pressure | Inverse variance weighted | 368 | -2.572 | 0.348 | 1.56E-13 |
| Whole body fat-free mass | systolic blood pressure | Simple mode | 368 | -2.242 | 1.290 | 8.30E-02 |
| Whole body fat-free mass | systolic blood pressure | Weighted mode | 368 | -3.606 | 2.405 | 1.35E-01 |
| Platelet distribution width | systolic blood pressure | MR Egger | 262 | 0.022 | 0.323 | 9.46E-01 |
| Platelet distribution width | systolic blood pressure | Weighted median | 262 | 0.145 | 0.129 | 2.62E-01 |
| Platelet distribution width | systolic blood pressure | Inverse variance weighted | 262 | 0.465 | 0.190 | 1.46E-02 |
| Platelet distribution width | systolic blood pressure | Simple mode | 262 | -0.348 | 0.277 | 2.11E-01 |
| Platelet distribution width | systolic blood pressure | Weighted mode | 262 | 0.010 | 0.121 | 9.35E-01 |
| IGF-1 | systolic blood pressure | MR Egger | 254 | -1.520 | 0.421 | 3.66E-04 |
| IGF-1 | systolic blood pressure | Weighted median | 254 | -0.673 | 0.198 | 6.66E-04 |
| IGF-1 | systolic blood pressure | Inverse variance weighted | 254 | -0.248 | 0.224 | 2.69E-01 |
| IGF-1 | systolic blood pressure | Simple mode | 254 | -0.948 | 0.394 | 1.69E-02 |
| IGF-1 | systolic blood pressure | Weighted mode | 254 | -1.027 | 0.185 | 7.61E-08 |
| White blood cell (leukocyte) count | systolic blood pressure | MR Egger | 250 | 0.953 | 0.613 | 1.21E-01 |
| White blood cell (leukocyte) count | systolic blood pressure | Weighted median | 250 | 0.194 | 0.187 | 2.99E-01 |
| White blood cell (leukocyte) count | systolic blood pressure | Inverse variance weighted | 250 | 0.690 | 0.269 | 1.04E-02 |
| White blood cell (leukocyte) count | systolic blood pressure | Simple mode | 250 | 0.031 | 0.395 | 9.38E-01 |
| White blood cell (leukocyte) count | systolic blood pressure | Weighted mode | 250 | 0.266 | 0.190 | 1.62E-01 |
| HDL cholesterol | systolic blood pressure | MR Egger | 202 | -0.211 | 0.352 | 5.50E-01 |
| HDL cholesterol | systolic blood pressure | Weighted median | 202 | -0.411 | 0.135 | 2.39E-03 |
| HDL cholesterol | systolic blood pressure | Inverse variance weighted | 202 | -0.672 | 0.243 | 5.60E-03 |
| HDL cholesterol | systolic blood pressure | Simple mode | 202 | -0.369 | 0.412 | 3.72E-01 |
| HDL cholesterol | systolic blood pressure | Weighted mode | 202 | -0.369 | 0.111 | 1.05E-03 |
| Triglycerides | systolic blood pressure | MR Egger | 177 | 0.372 | 0.360 | 3.03E-01 |
| Triglycerides | systolic blood pressure | Weighted median | 177 | 0.696 | 0.148 | 2.70E-06 |
| Triglycerides | systolic blood pressure | Inverse variance weighted | 177 | 1.241 | 0.256 | 1.22E-06 |
| Triglycerides | systolic blood pressure | Simple mode | 177 | 1.124 | 0.572 | 5.09E-02 |
| Triglycerides | systolic blood pressure | Weighted mode | 177 | 0.619 | 0.131 | 4.51E-06 |
| Reticulocyte count | systolic blood pressure | MR Egger | 221 | 0.538 | 0.446 | 2.30E-01 |
| Reticulocyte count | systolic blood pressure | Weighted median | 221 | 0.240 | 0.163 | 1.40E-01 |
| Reticulocyte count | systolic blood pressure | Inverse variance weighted | 221 | 0.833 | 0.257 | 1.17E-03 |
| Reticulocyte count | systolic blood pressure | Simple mode | 221 | -0.043 | 0.372 | 9.08E-01 |
| Reticulocyte count | systolic blood pressure | Weighted mode | 221 | 0.083 | 0.150 | 5.81E-01 |
| Waist-to-hip ratio | systolic blood pressure | MR Egger | 26 | -2.211 | 4.019 | 5.87E-01 |
| Waist-to-hip ratio | systolic blood pressure | Weighted median | 26 | 2.866 | 0.550 | 1.86E-07 |
| Waist-to-hip ratio | systolic blood pressure | Inverse variance weighted | 26 | 2.451 | 0.923 | 7.88E-03 |
| Waist-to-hip ratio | systolic blood pressure | Simple mode | 26 | 3.935 | 0.794 | 4.18E-05 |
| Waist-to-hip ratio | systolic blood pressure | Weighted mode | 26 | 3.546 | 0.903 | 5.96E-04 |
| Apoliprotein A | systolic blood pressure | MR Egger | 188 | 0.128 | 0.396 | 7.47E-01 |
| Apoliprotein A | systolic blood pressure | Weighted median | 188 | -0.310 | 0.155 | 4.57E-02 |
| Apoliprotein A | systolic blood pressure | Inverse variance weighted | 188 | -0.171 | 0.265 | 5.18E-01 |
| Apoliprotein A | systolic blood pressure | Simple mode | 188 | -0.254 | 0.340 | 4.56E-01 |
| Apoliprotein A | systolic blood pressure | Weighted mode | 188 | -0.254 | 0.127 | 4.66E-02 |
| Monocyte count | systolic blood pressure | MR Egger | 239 | 0.067 | 0.384 | 8.62E-01 |
| Monocyte count | systolic blood pressure | Weighted median | 239 | 0.086 | 0.165 | 6.03E-01 |
| Monocyte count | systolic blood pressure | Inverse variance weighted | 239 | 0.359 | 0.212 | 9.07E-02 |
| Monocyte count | systolic blood pressure | Simple mode | 239 | 0.165 | 0.322 | 6.08E-01 |
| Monocyte count | systolic blood pressure | Weighted mode | 239 | 0.165 | 0.141 | 2.41E-01 |
| Fasting insulin | systolic blood pressure | MR Egger | 14 | -9.706 | 10.896 | 3.91E-01 |
| Fasting insulin | systolic blood pressure | Weighted median | 14 | 6.489 | 1.420 | 4.92E-06 |
| Fasting insulin | systolic blood pressure | Inverse variance weighted | 14 | 7.661 | 2.146 | 3.57E-04 |
| Fasting insulin | systolic blood pressure | Simple mode | 14 | 12.690 | 2.057 | 3.38E-05 |
| Fasting insulin | systolic blood pressure | Weighted mode | 14 | 12.435 | 2.645 | 4.14E-04 |
| Weight | systolic blood pressure | MR Egger | 307 | -2.012 | 0.945 | 3.40E-02 |
| Weight | systolic blood pressure | Weighted median | 307 | -0.704 | 0.247 | 4.35E-03 |
| Weight | systolic blood pressure | Inverse variance weighted | 307 | -1.488 | 0.341 | 1.27E-05 |
| Weight | systolic blood pressure | Simple mode | 307 | -1.024 | 1.092 | 3.49E-01 |
| Weight | systolic blood pressure | Weighted mode | 307 | 0.128 | 0.510 | 8.01E-01 |
| Birth weight of first child | systolic blood pressure | MR Egger | 39 | -1.566 | 3.589 | 6.65E-01 |
| Birth weight of first child | systolic blood pressure | Weighted median | 39 | -1.457 | 0.308 | 2.23E-06 |
| Birth weight of first child | systolic blood pressure | Inverse variance weighted | 39 | -3.492 | 0.922 | 1.51E-04 |
| Birth weight of first child | systolic blood pressure | Simple mode | 39 | -1.392 | 0.424 | 2.22E-03 |
| Birth weight of first child | systolic blood pressure | Weighted mode | 39 | -1.319 | 0.398 | 2.00E-03 |
| Basal metabolic rate | systolic blood pressure | MR Egger | 338 | -3.006 | 0.908 | 1.03E-03 |
| Basal metabolic rate | systolic blood pressure | Weighted median | 338 | -1.849 | 0.267 | 4.27E-12 |
| Basal metabolic rate | systolic blood pressure | Inverse variance weighted | 338 | -2.235 | 0.350 | 1.61E-10 |
| Basal metabolic rate | systolic blood pressure | Simple mode | 338 | -2.154 | 1.400 | 1.25E-01 |
| Basal metabolic rate | systolic blood pressure | Weighted mode | 338 | -0.667 | 2.067 | 7.47E-01 |
| Standing height | systolic blood pressure | MR Egger | 526 | -0.662 | 0.474 | 1.63E-01 |
| Standing height | systolic blood pressure | Weighted median | 526 | -0.386 | 0.163 | 1.78E-02 |
| Standing height | systolic blood pressure | Inverse variance weighted | 526 | -0.956 | 0.212 | 6.30E-06 |
| Standing height | systolic blood pressure | Simple mode | 526 | 0.814 | 0.793 | 3.05E-01 |
| Standing height | systolic blood pressure | Weighted mode | 526 | 1.372 | 0.413 | 9.46E-04 |
| Total bilirubin | systolic blood pressure | MR Egger | 92 | -0.062 | 0.116 | 5.96E-01 |
| Total bilirubin | systolic blood pressure | Weighted median | 92 | -0.051 | 0.042 | 2.26E-01 |
| Total bilirubin | systolic blood pressure | Inverse variance weighted | 92 | -0.047 | 0.109 | 6.66E-01 |
| Total bilirubin | systolic blood pressure | Simple mode | 92 | 0.260 | 0.635 | 6.83E-01 |
| Total bilirubin | systolic blood pressure | Weighted mode | 92 | -0.044 | 0.050 | 3.85E-01 |
| Drive faster than motorway speed limit | systolic blood pressure | MR Egger | 11 | 1.364 | 18.346 | 9.42E-01 |
| Drive faster than motorway speed limit | systolic blood pressure | Weighted median | 11 | -2.894 | 1.583 | 6.74E-02 |
| Drive faster than motorway speed limit | systolic blood pressure | Inverse variance weighted | 11 | -7.126 | 3.152 | 2.38E-02 |
| Drive faster than motorway speed limit | systolic blood pressure | Simple mode | 11 | -4.037 | 2.520 | 1.40E-01 |
| Drive faster than motorway speed limit | systolic blood pressure | Weighted mode | 11 | -2.792 | 1.657 | 1.23E-01 |
| Overall health rating | systolic blood pressure | MR Egger | 49 | -12.129 | 7.766 | 1.25E-01 |
| Overall health rating | systolic blood pressure | Weighted median | 49 | 2.262 | 0.734 | 2.05E-03 |
| Overall health rating | systolic blood pressure | Inverse variance weighted | 49 | 2.211 | 1.466 | 1.31E-01 |
| Overall health rating | systolic blood pressure | Simple mode | 49 | 1.841 | 1.159 | 1.19E-01 |
| Overall health rating | systolic blood pressure | Weighted mode | 49 | 1.841 | 1.124 | 1.08E-01 |
| Phosphate | systolic blood pressure | MR Egger | 123 | -0.466 | 0.593 | 4.33E-01 |
| Phosphate | systolic blood pressure | Weighted median | 123 | -0.296 | 0.196 | 1.31E-01 |
| Phosphate | systolic blood pressure | Inverse variance weighted | 123 | -0.135 | 0.351 | 7.01E-01 |
| Phosphate | systolic blood pressure | Simple mode | 123 | -0.062 | 0.436 | 8.86E-01 |
| Phosphate | systolic blood pressure | Weighted mode | 123 | -0.249 | 0.165 | 1.33E-01 |
| Cigarettes per Day | systolic blood pressure | MR Egger | 22 | 0.208 | 0.460 | 6.56E-01 |
| Cigarettes per Day | systolic blood pressure | Weighted median | 22 | 0.334 | 0.166 | 4.42E-02 |
| Cigarettes per Day | systolic blood pressure | Inverse variance weighted | 22 | 0.303 | 0.259 | 2.42E-01 |
| Cigarettes per Day | systolic blood pressure | Simple mode | 22 | 0.919 | 0.454 | 5.59E-02 |
| Cigarettes per Day | systolic blood pressure | Weighted mode | 22 | 0.306 | 0.159 | 6.83E-02 |
| Mean time to correctly identify matches | systolic blood pressure | MR Egger | 28 | 10.362 | 8.658 | 2.42E-01 |
| Mean time to correctly identify matches | systolic blood pressure | Weighted median | 28 | 1.117 | 0.828 | 1.77E-01 |
| Mean time to correctly identify matches | systolic blood pressure | Inverse variance weighted | 28 | 1.911 | 1.463 | 1.92E-01 |
| Mean time to correctly identify matches | systolic blood pressure | Simple mode | 28 | 3.397 | 2.008 | 1.02E-01 |
| Mean time to correctly identify matches | systolic blood pressure | Weighted mode | 28 | 2.523 | 1.738 | 1.58E-01 |
| Mean corpuscular haemoglobin | systolic blood pressure | MR Egger | 263 | 0.151 | 0.237 | 5.25E-01 |
| Mean corpuscular haemoglobin | systolic blood pressure | Weighted median | 263 | -0.267 | 0.131 | 4.16E-02 |
| Mean corpuscular haemoglobin | systolic blood pressure | Inverse variance weighted | 263 | -0.050 | 0.151 | 7.39E-01 |
| Mean corpuscular haemoglobin | systolic blood pressure | Simple mode | 263 | -0.377 | 0.300 | 2.10E-01 |
| Mean corpuscular haemoglobin | systolic blood pressure | Weighted mode | 263 | -0.175 | 0.109 | 1.10E-01 |
| Albumin | systolic blood pressure | MR Egger | 150 | 0.823 | 0.722 | 2.56E-01 |
| Albumin | systolic blood pressure | Weighted median | 150 | 1.116 | 0.250 | 8.17E-06 |
| Albumin | systolic blood pressure | Inverse variance weighted | 150 | 1.208 | 0.392 | 2.08E-03 |
| Albumin | systolic blood pressure | Simple mode | 150 | 1.289 | 0.710 | 7.17E-02 |
| Albumin | systolic blood pressure | Weighted mode | 150 | 1.188 | 0.364 | 1.38E-03 |
| Urinary sodium-potassium ratio | systolic blood pressure | MR Egger | 23 | -8.394 | 6.286 | 1.96E-01 |
| Urinary sodium-potassium ratio | systolic blood pressure | Weighted median | 23 | 1.684 | 0.946 | 7.51E-02 |
| Urinary sodium-potassium ratio | systolic blood pressure | Inverse variance weighted | 23 | 2.260 | 1.596 | 1.57E-01 |
| Urinary sodium-potassium ratio | systolic blood pressure | Simple mode | 23 | 2.707 | 1.786 | 1.44E-01 |
| Urinary sodium-potassium ratio | systolic blood pressure | Weighted mode | 23 | 3.568 | 1.561 | 3.23E-02 |
| Haematocrit percentage | systolic blood pressure | MR Egger | 200 | -0.308 | 0.848 | 7.16E-01 |
| Haematocrit percentage | systolic blood pressure | Weighted median | 200 | -0.537 | 0.279 | 5.44E-02 |
| Haematocrit percentage | systolic blood pressure | Inverse variance weighted | 200 | -0.165 | 0.432 | 7.02E-01 |
| Haematocrit percentage | systolic blood pressure | Simple mode | 200 | -1.182 | 0.788 | 1.35E-01 |
| Haematocrit percentage | systolic blood pressure | Weighted mode | 200 | -0.840 | 0.457 | 6.75E-02 |
| Sitting height | systolic blood pressure | MR Egger | 384 | -0.897 | 0.666 | 1.79E-01 |
| Sitting height | systolic blood pressure | Weighted median | 384 | -0.539 | 0.201 | 7.22E-03 |
| Sitting height | systolic blood pressure | Inverse variance weighted | 384 | -0.759 | 0.254 | 2.79E-03 |
| Sitting height | systolic blood pressure | Simple mode | 384 | -1.707 | 0.833 | 4.13E-02 |
| Sitting height | systolic blood pressure | Weighted mode | 384 | -1.051 | 1.047 | 3.16E-01 |
| Monocyte percentage | systolic blood pressure | MR Egger | 223 | 0.169 | 0.294 | 5.66E-01 |
| Monocyte percentage | systolic blood pressure | Weighted median | 223 | 0.071 | 0.150 | 6.33E-01 |
| Monocyte percentage | systolic blood pressure | Inverse variance weighted | 223 | -0.046 | 0.160 | 7.72E-01 |
| Monocyte percentage | systolic blood pressure | Simple mode | 223 | 0.087 | 0.318 | 7.85E-01 |
| Monocyte percentage | systolic blood pressure | Weighted mode | 223 | 0.144 | 0.143 | 3.17E-01 |
| SHBG | systolic blood pressure | MR Egger | 207 | 0.094 | 0.347 | 7.86E-01 |
| SHBG | systolic blood pressure | Weighted median | 207 | 0.263 | 0.151 | 8.09E-02 |
| SHBG | systolic blood pressure | Inverse variance weighted | 207 | -0.938 | 0.233 | 5.65E-05 |
| SHBG | systolic blood pressure | Simple mode | 207 | -1.710 | 0.632 | 7.44E-03 |
| SHBG | systolic blood pressure | Weighted mode | 207 | 0.088 | 0.144 | 5.40E-01 |
| Lymphocyte percentage | systolic blood pressure | MR Egger | 206 | 0.129 | 0.719 | 8.57E-01 |
| Lymphocyte percentage | systolic blood pressure | Weighted median | 206 | -0.109 | 0.221 | 6.21E-01 |
| Lymphocyte percentage | systolic blood pressure | Inverse variance weighted | 206 | 0.118 | 0.299 | 6.94E-01 |
| Lymphocyte percentage | systolic blood pressure | Simple mode | 206 | 0.938 | 0.561 | 9.62E-02 |
| Lymphocyte percentage | systolic blood pressure | Weighted mode | 206 | 0.098 | 0.290 | 7.37E-01 |
| C-reactive protein | systolic blood pressure | MR Egger | 147 | -0.158 | 0.309 | 6.11E-01 |
| C-reactive protein | systolic blood pressure | Weighted median | 147 | -0.436 | 0.137 | 1.45E-03 |
| C-reactive protein | systolic blood pressure | Inverse variance weighted | 147 | -0.118 | 0.216 | 5.84E-01 |
| C-reactive protein | systolic blood pressure | Simple mode | 147 | 0.311 | 0.484 | 5.21E-01 |
| C-reactive protein | systolic blood pressure | Weighted mode | 147 | -0.331 | 0.115 | 4.74E-03 |
| Cystatin C | systolic blood pressure | MR Egger | 239 | -0.064 | 0.356 | 8.57E-01 |
| Cystatin C | systolic blood pressure | Weighted median | 239 | -0.294 | 0.102 | 4.02E-03 |
| Cystatin C | systolic blood pressure | Inverse variance weighted | 239 | 0.163 | 0.261 | 5.31E-01 |
| Cystatin C | systolic blood pressure | Simple mode | 239 | 1.873 | 0.890 | 3.63E-02 |
| Cystatin C | systolic blood pressure | Weighted mode | 239 | -0.349 | 0.098 | 4.53E-04 |
| Eosinophill percentage | systolic blood pressure | MR Egger | 236 | 1.197 | 0.482 | 1.37E-02 |
| Eosinophill percentage | systolic blood pressure | Weighted median | 236 | 0.107 | 0.172 | 5.34E-01 |
| Eosinophill percentage | systolic blood pressure | Inverse variance weighted | 236 | 0.460 | 0.236 | 5.13E-02 |
| Eosinophill percentage | systolic blood pressure | Simple mode | 236 | -0.140 | 0.412 | 7.34E-01 |
| Eosinophill percentage | systolic blood pressure | Weighted mode | 236 | -0.041 | 0.215 | 8.49E-01 |
| Red blood cell (erythrocyte) distribution width | systolic blood pressure | MR Egger | 234 | -0.084 | 0.328 | 7.99E-01 |
| Red blood cell (erythrocyte) distribution width | systolic blood pressure | Weighted median | 234 | 0.251 | 0.136 | 6.48E-02 |
| Red blood cell (erythrocyte) distribution width | systolic blood pressure | Inverse variance weighted | 234 | 0.002 | 0.186 | 9.91E-01 |
| Red blood cell (erythrocyte) distribution width | systolic blood pressure | Simple mode | 234 | -0.117 | 0.306 | 7.02E-01 |
| Red blood cell (erythrocyte) distribution width | systolic blood pressure | Weighted mode | 234 | 0.393 | 0.148 | 8.29E-03 |
| Neutrophill percentage | systolic blood pressure | MR Egger | 201 | -0.888 | 0.765 | 2.47E-01 |
| Neutrophill percentage | systolic blood pressure | Weighted median | 201 | 0.178 | 0.216 | 4.09E-01 |
| Neutrophill percentage | systolic blood pressure | Inverse variance weighted | 201 | -0.082 | 0.298 | 7.83E-01 |
| Neutrophill percentage | systolic blood pressure | Simple mode | 201 | -0.300 | 0.585 | 6.09E-01 |
| Neutrophill percentage | systolic blood pressure | Weighted mode | 201 | 0.278 | 0.334 | 4.06E-01 |
| Getting up in morning | systolic blood pressure | MR Egger | 32 | 1.535 | 3.049 | 6.18E-01 |
| Getting up in morning | systolic blood pressure | Weighted median | 32 | 1.204 | 0.725 | 9.68E-02 |
| Getting up in morning | systolic blood pressure | Inverse variance weighted | 32 | 0.966 | 0.945 | 3.06E-01 |
| Getting up in morning | systolic blood pressure | Simple mode | 32 | 0.992 | 1.315 | 4.56E-01 |
| Getting up in morning | systolic blood pressure | Weighted mode | 32 | 0.853 | 1.177 | 4.74E-01 |
| Platelet crit | systolic blood pressure | MR Egger | 291 | 1.788 | 0.419 | 2.65E-05 |
| Platelet crit | systolic blood pressure | Weighted median | 291 | 0.162 | 0.167 | 3.33E-01 |
| Platelet crit | systolic blood pressure | Inverse variance weighted | 291 | 0.668 | 0.225 | 2.95E-03 |
| Platelet crit | systolic blood pressure | Simple mode | 291 | 0.025 | 0.395 | 9.49E-01 |
| Platelet crit | systolic blood pressure | Weighted mode | 291 | -0.079 | 0.188 | 6.76E-01 |
| Body fat | systolic blood pressure | MR Egger | 9 | 1.512 | 6.487 | 8.22E-01 |
| Body fat | systolic blood pressure | Weighted median | 9 | -1.553 | 0.701 | 2.66E-02 |
| Body fat | systolic blood pressure | Inverse variance weighted | 9 | -2.094 | 1.387 | 1.31E-01 |
| Body fat | systolic blood pressure | Simple mode | 9 | -3.349 | 1.782 | 9.70E-02 |
| Body fat | systolic blood pressure | Weighted mode | 9 | -0.533 | 0.622 | 4.17E-01 |
| Morning/evening person (chronotype) | systolic blood pressure | MR Egger | 71 | -3.486 | 1.525 | 2.53E-02 |
| Morning/evening person (chronotype) | systolic blood pressure | Weighted median | 71 | -0.302 | 0.414 | 4.66E-01 |
| Morning/evening person (chronotype) | systolic blood pressure | Inverse variance weighted | 71 | -0.496 | 0.501 | 3.22E-01 |
| Morning/evening person (chronotype) | systolic blood pressure | Simple mode | 71 | 0.095 | 1.092 | 9.31E-01 |
| Morning/evening person (chronotype) | systolic blood pressure | Weighted mode | 71 | -0.333 | 0.999 | 7.40E-01 |
| LDL direct | systolic blood pressure | MR Egger | 116 | 0.805 | 0.588 | 1.74E-01 |
| LDL direct | systolic blood pressure | Weighted median | 116 | 0.675 | 0.185 | 2.74E-04 |
| LDL direct | systolic blood pressure | Inverse variance weighted | 116 | 0.068 | 0.390 | 8.62E-01 |
| LDL direct | systolic blood pressure | Simple mode | 116 | 0.907 | 0.366 | 1.48E-02 |
| LDL direct | systolic blood pressure | Weighted mode | 116 | 0.674 | 0.165 | 8.32E-05 |
| Direct bilirubin | systolic blood pressure | MR Egger | 57 | -0.061 | 0.146 | 6.79E-01 |
| Direct bilirubin | systolic blood pressure | Weighted median | 57 | -0.051 | 0.051 | 3.20E-01 |
| Direct bilirubin | systolic blood pressure | Inverse variance weighted | 57 | -0.060 | 0.133 | 6.53E-01 |
| Direct bilirubin | systolic blood pressure | Simple mode | 57 | -1.945 | 0.845 | 2.51E-02 |
| Direct bilirubin | systolic blood pressure | Weighted mode | 57 | -0.016 | 0.056 | 7.78E-01 |
| Forced vital capacity (FVC) | systolic blood pressure | MR Egger | 191 | -2.635 | 1.283 | 4.14E-02 |
| Forced vital capacity (FVC) | systolic blood pressure | Weighted median | 191 | -1.793 | 0.337 | 1.00E-07 |
| Forced vital capacity (FVC) | systolic blood pressure | Inverse variance weighted | 191 | -2.122 | 0.435 | 1.07E-06 |
| Forced vital capacity (FVC) | systolic blood pressure | Simple mode | 191 | -3.644 | 1.228 | 3.39E-03 |
| Forced vital capacity (FVC) | systolic blood pressure | Weighted mode | 191 | -2.549 | 0.927 | 6.56E-03 |
| Waist circumference | systolic blood pressure | MR Egger | 195 | -2.090 | 1.233 | 9.17E-02 |
| Waist circumference | systolic blood pressure | Weighted median | 195 | 0.080 | 0.313 | 7.99E-01 |
| Waist circumference | systolic blood pressure | Inverse variance weighted | 195 | -0.617 | 0.390 | 1.14E-01 |
| Waist circumference | systolic blood pressure | Simple mode | 195 | 0.582 | 0.815 | 4.76E-01 |
| Waist circumference | systolic blood pressure | Weighted mode | 195 | 0.305 | 0.472 | 5.19E-01 |
| Neutrophill count | systolic blood pressure | MR Egger | 206 | -0.149 | 0.755 | 8.44E-01 |
| Neutrophill count | systolic blood pressure | Weighted median | 206 | 0.141 | 0.197 | 4.75E-01 |
| Neutrophill count | systolic blood pressure | Inverse variance weighted | 206 | 0.406 | 0.315 | 1.97E-01 |
| Neutrophill count | systolic blood pressure | Simple mode | 206 | 0.550 | 0.451 | 2.24E-01 |
| Neutrophill count | systolic blood pressure | Weighted mode | 206 | 0.211 | 0.233 | 3.66E-01 |
| Mean corpuscular volume | systolic blood pressure | MR Egger | 265 | 0.333 | 0.249 | 1.82E-01 |
| Mean corpuscular volume | systolic blood pressure | Weighted median | 265 | -0.293 | 0.128 | 2.18E-02 |
| Mean corpuscular volume | systolic blood pressure | Inverse variance weighted | 265 | -0.157 | 0.150 | 2.97E-01 |
| Mean corpuscular volume | systolic blood pressure | Simple mode | 265 | -0.315 | 0.327 | 3.36E-01 |
| Mean corpuscular volume | systolic blood pressure | Weighted mode | 265 | -0.130 | 0.134 | 3.31E-01 |
| Forced expiratory volume in 1-second (FEV1) | systolic blood pressure | MR Egger | 139 | -2.734 | 1.774 | 1.25E-01 |
| Forced expiratory volume in 1-second (FEV1) | systolic blood pressure | Weighted median | 139 | -1.627 | 0.358 | 5.61E-06 |
| Forced expiratory volume in 1-second (FEV1) | systolic blood pressure | Inverse variance weighted | 139 | -1.837 | 0.523 | 4.46E-04 |
| Forced expiratory volume in 1-second (FEV1) | systolic blood pressure | Simple mode | 139 | -3.003 | 0.964 | 2.25E-03 |
| Forced expiratory volume in 1-second (FEV1) | systolic blood pressure | Weighted mode | 139 | -2.469 | 0.730 | 9.29E-04 |
| Cholesterol | systolic blood pressure | MR Egger | 122 | 0.577 | 0.551 | 2.97E-01 |
| Cholesterol | systolic blood pressure | Weighted median | 122 | 0.450 | 0.197 | 2.25E-02 |
| Cholesterol | systolic blood pressure | Inverse variance weighted | 122 | 0.042 | 0.335 | 9.00E-01 |
| Cholesterol | systolic blood pressure | Simple mode | 122 | 0.637 | 0.376 | 9.34E-02 |
| Cholesterol | systolic blood pressure | Weighted mode | 122 | 0.637 | 0.175 | 4.14E-04 |
| Sodium in urine | systolic blood pressure | MR Egger | 28 | -4.608 | 7.325 | 5.35E-01 |
| Sodium in urine | systolic blood pressure | Weighted median | 28 | 1.488 | 0.871 | 8.76E-02 |
| Sodium in urine | systolic blood pressure | Inverse variance weighted | 28 | 1.268 | 1.308 | 3.32E-01 |
| Sodium in urine | systolic blood pressure | Simple mode | 28 | 2.656 | 1.880 | 1.69E-01 |
| Sodium in urine | systolic blood pressure | Weighted mode | 28 | 2.739 | 1.715 | 1.22E-01 |
| Daytime dozing / sleeping (narcolepsy) | systolic blood pressure | MR Egger | 19 | 12.236 | 11.144 | 2.88E-01 |
| Daytime dozing / sleeping (narcolepsy) | systolic blood pressure | Weighted median | 19 | 1.473 | 1.513 | 3.30E-01 |
| Daytime dozing / sleeping (narcolepsy) | systolic blood pressure | Inverse variance weighted | 19 | 4.395 | 2.236 | 4.94E-02 |
| Daytime dozing / sleeping (narcolepsy) | systolic blood pressure | Simple mode | 19 | -0.203 | 2.209 | 9.28E-01 |
| Daytime dozing / sleeping (narcolepsy) | systolic blood pressure | Weighted mode | 19 | -0.427 | 2.076 | 8.39E-01 |
| Pulse rate automated reading | systolic blood pressure | MR Egger | 182 | -1.036 | 0.837 | 2.18E-01 |
| Pulse rate automated reading | systolic blood pressure | Weighted median | 182 | -1.066 | 0.233 | 4.90E-06 |
| Pulse rate automated reading | systolic blood pressure | Inverse variance weighted | 182 | -1.057 | 0.367 | 4.02E-03 |
| Pulse rate automated reading | systolic blood pressure | Simple mode | 182 | -1.455 | 0.499 | 3.99E-03 |
| Pulse rate automated reading | systolic blood pressure | Weighted mode | 182 | -1.212 | 0.279 | 2.27E-05 |
| Mean platelet (thrombocyte) volume | systolic blood pressure | MR Egger | 321 | 0.014 | 0.174 | 9.35E-01 |
| Mean platelet (thrombocyte) volume | systolic blood pressure | Weighted median | 321 | -0.145 | 0.094 | 1.21E-01 |
| Mean platelet (thrombocyte) volume | systolic blood pressure | Inverse variance weighted | 321 | -0.051 | 0.118 | 6.65E-01 |
| Mean platelet (thrombocyte) volume | systolic blood pressure | Simple mode | 321 | 0.056 | 0.212 | 7.93E-01 |
| Mean platelet (thrombocyte) volume | systolic blood pressure | Weighted mode | 321 | -0.061 | 0.079 | 4.41E-01 |
| Testosterone | systolic blood pressure | MR Egger | 72 | 2.300 | 1.797 | 2.05E-01 |
| Testosterone | systolic blood pressure | Weighted median | 72 | 1.632 | 0.708 | 2.11E-02 |
| Testosterone | systolic blood pressure | Inverse variance weighted | 72 | -1.055 | 1.035 | 3.08E-01 |
| Testosterone | systolic blood pressure | Simple mode | 72 | -1.217 | 1.438 | 4.00E-01 |
| Testosterone | systolic blood pressure | Weighted mode | 72 | 2.845 | 0.483 | 1.16E-07 |
| Mean sphered cell volume | systolic blood pressure | MR Egger | 248 | 0.296 | 0.312 | 3.44E-01 |
| Mean sphered cell volume | systolic blood pressure | Weighted median | 248 | 0.185 | 0.134 | 1.66E-01 |
| Mean sphered cell volume | systolic blood pressure | Inverse variance weighted | 248 | -0.285 | 0.180 | 1.12E-01 |
| Mean sphered cell volume | systolic blood pressure | Simple mode | 248 | 0.099 | 0.288 | 7.32E-01 |
| Mean sphered cell volume | systolic blood pressure | Weighted mode | 248 | 0.283 | 0.131 | 3.18E-02 |
| Fluid intelligence score | systolic blood pressure | MR Egger | 37 | 2.357 | 1.445 | 1.12E-01 |
| Fluid intelligence score | systolic blood pressure | Weighted median | 37 | -0.791 | 0.175 | 5.96E-06 |
| Fluid intelligence score | systolic blood pressure | Inverse variance weighted | 37 | -0.267 | 0.359 | 4.57E-01 |
| Fluid intelligence score | systolic blood pressure | Simple mode | 37 | -0.944 | 0.339 | 8.39E-03 |
| Fluid intelligence score | systolic blood pressure | Weighted mode | 37 | -0.915 | 0.321 | 7.18E-03 |
| Mean reticulocyte volume | systolic blood pressure | MR Egger | 242 | 0.382 | 0.316 | 2.29E-01 |
| Mean reticulocyte volume | systolic blood pressure | Weighted median | 242 | 0.068 | 0.127 | 5.92E-01 |
| Mean reticulocyte volume | systolic blood pressure | Inverse variance weighted | 242 | -0.318 | 0.174 | 6.77E-02 |
| Mean reticulocyte volume | systolic blood pressure | Simple mode | 242 | -0.163 | 0.279 | 5.58E-01 |
| Mean reticulocyte volume | systolic blood pressure | Weighted mode | 242 | 0.158 | 0.130 | 2.26E-01 |
| Creatinine | systolic blood pressure | MR Egger | 244 | 0.049 | 0.858 | 9.55E-01 |
| Creatinine | systolic blood pressure | Weighted median | 244 | -0.444 | 0.286 | 1.20E-01 |
| Creatinine | systolic blood pressure | Inverse variance weighted | 244 | -0.419 | 0.369 | 2.57E-01 |
| Creatinine | systolic blood pressure | Simple mode | 244 | -0.041 | 1.240 | 9.74E-01 |
| Creatinine | systolic blood pressure | Weighted mode | 244 | -0.824 | 0.671 | 2.20E-01 |
| High light scatter reticulocyte count | systolic blood pressure | MR Egger | 234 | 0.584 | 0.435 | 1.81E-01 |
| High light scatter reticulocyte count | systolic blood pressure | Weighted median | 234 | 0.280 | 0.172 | 1.04E-01 |
| High light scatter reticulocyte count | systolic blood pressure | Inverse variance weighted | 234 | 0.709 | 0.243 | 3.45E-03 |
| High light scatter reticulocyte count | systolic blood pressure | Simple mode | 234 | 0.347 | 0.373 | 3.53E-01 |
| High light scatter reticulocyte count | systolic blood pressure | Weighted mode | 234 | 0.347 | 0.142 | 1.50E-02 |
| Alcohol intake frequency. | systolic blood pressure | MR Egger | 38 | -2.149 | 1.333 | 1.16E-01 |
| Alcohol intake frequency. | systolic blood pressure | Weighted median | 38 | -1.540 | 0.382 | 5.56E-05 |
| Alcohol intake frequency. | systolic blood pressure | Inverse variance weighted | 38 | -0.920 | 0.707 | 1.93E-01 |
| Alcohol intake frequency. | systolic blood pressure | Simple mode | 38 | 0.750 | 0.922 | 4.21E-01 |
| Alcohol intake frequency. | systolic blood pressure | Weighted mode | 38 | -1.759 | 0.288 | 4.45E-07 |
| Impedance of whole body | systolic blood pressure | MR Egger | 319 | 3.730 | 1.101 | 7.96E-04 |
| Impedance of whole body | systolic blood pressure | Weighted median | 319 | 1.647 | 0.248 | 3.03E-11 |
| Impedance of whole body | systolic blood pressure | Inverse variance weighted | 319 | 2.016 | 0.392 | 2.76E-07 |
| Impedance of whole body | systolic blood pressure | Simple mode | 319 | 1.990 | 0.882 | 2.48E-02 |
| Impedance of whole body | systolic blood pressure | Weighted mode | 319 | 1.234 | 0.895 | 1.69E-01 |
| Past tobacco smoking | systolic blood pressure | MR Egger | 39 | 6.650 | 4.312 | 1.32E-01 |
| Past tobacco smoking | systolic blood pressure | Weighted median | 39 | 2.678 | 0.507 | 1.27E-07 |
| Past tobacco smoking | systolic blood pressure | Inverse variance weighted | 39 | 2.756 | 1.017 | 6.75E-03 |
| Past tobacco smoking | systolic blood pressure | Simple mode | 39 | 2.307 | 0.898 | 1.43E-02 |
| Past tobacco smoking | systolic blood pressure | Weighted mode | 39 | 2.586 | 0.767 | 1.72E-03 |
| Alanine aminotransferase | systolic blood pressure | MR Egger | 153 | 0.050 | 0.830 | 9.52E-01 |
| Alanine aminotransferase | systolic blood pressure | Weighted median | 153 | 0.481 | 0.269 | 7.32E-02 |
| Alanine aminotransferase | systolic blood pressure | Inverse variance weighted | 153 | 1.733 | 0.403 | 1.72E-05 |
| Alanine aminotransferase | systolic blood pressure | Simple mode | 153 | 0.366 | 0.951 | 7.01E-01 |
| Alanine aminotransferase | systolic blood pressure | Weighted mode | 153 | 0.646 | 0.407 | 1.15E-01 |
| Heart rate | systolic blood pressure | MR Egger | 14 | -0.264 | 0.204 | 2.19E-01 |
| Heart rate | systolic blood pressure | Weighted median | 14 | -0.120 | 0.033 | 2.19E-04 |
| Heart rate | systolic blood pressure | Inverse variance weighted | 14 | -0.117 | 0.050 | 1.88E-02 |
| Heart rate | systolic blood pressure | Simple mode | 14 | -0.120 | 0.052 | 3.65E-02 |
| Heart rate | systolic blood pressure | Weighted mode | 14 | -0.120 | 0.053 | 4.06E-02 |
| Reticulocyte percentage | systolic blood pressure | MR Egger | 213 | 0.352 | 0.427 | 4.11E-01 |
| Reticulocyte percentage | systolic blood pressure | Weighted median | 213 | 0.225 | 0.160 | 1.60E-01 |
| Reticulocyte percentage | systolic blood pressure | Inverse variance weighted | 213 | 0.640 | 0.243 | 8.46E-03 |
| Reticulocyte percentage | systolic blood pressure | Simple mode | 213 | -0.234 | 0.365 | 5.23E-01 |
| Reticulocyte percentage | systolic blood pressure | Weighted mode | 213 | 0.120 | 0.137 | 3.83E-01 |
| telomere length | systolic blood pressure | MR Egger | 118 | 2.452 | 0.722 | 9.29E-04 |
| telomere length | systolic blood pressure | Weighted median | 118 | 2.375 | 0.274 | 4.45E-18 |
| telomere length | systolic blood pressure | Inverse variance weighted | 118 | 1.670 | 0.404 | 3.48E-05 |
| telomere length | systolic blood pressure | Simple mode | 118 | 2.103 | 0.594 | 5.70E-04 |
| telomere length | systolic blood pressure | Weighted mode | 118 | 2.219 | 0.271 | 3.83E-13 |
| Urate | systolic blood pressure | MR Egger | 187 | -0.020 | 0.377 | 9.58E-01 |
| Urate | systolic blood pressure | Weighted median | 187 | -0.053 | 0.119 | 6.55E-01 |
| Urate | systolic blood pressure | Inverse variance weighted | 187 | 0.919 | 0.295 | 1.80E-03 |
| Urate | systolic blood pressure | Simple mode | 187 | 2.470 | 0.778 | 1.77E-03 |
| Urate | systolic blood pressure | Weighted mode | 187 | -0.037 | 0.104 | 7.21E-01 |
| Birth weight | systolic blood pressure | MR Egger | 69 | 1.579 | 2.507 | 5.31E-01 |
| Birth weight | systolic blood pressure | Weighted median | 69 | -0.607 | 0.389 | 1.19E-01 |
| Birth weight | systolic blood pressure | Inverse variance weighted | 69 | -1.723 | 0.714 | 1.58E-02 |
| Birth weight | systolic blood pressure | Simple mode | 69 | -0.920 | 1.145 | 4.24E-01 |
| Birth weight | systolic blood pressure | Weighted mode | 69 | -0.475 | 1.057 | 6.54E-01 |
| Haemoglobin concentration | systolic blood pressure | MR Egger | 206 | -0.093 | 0.766 | 9.03E-01 |
| Haemoglobin concentration | systolic blood pressure | Weighted median | 206 | -0.714 | 0.280 | 1.06E-02 |
| Haemoglobin concentration | systolic blood pressure | Inverse variance weighted | 206 | 0.226 | 0.411 | 5.83E-01 |
| Haemoglobin concentration | systolic blood pressure | Simple mode | 206 | -1.393 | 0.826 | 9.33E-02 |
| Haemoglobin concentration | systolic blood pressure | Weighted mode | 206 | -0.634 | 0.371 | 8.92E-02 |
| Adiponectin | systolic blood pressure | MR Egger | 10 | -0.321 | 1.293 | 8.10E-01 |
| Adiponectin | systolic blood pressure | Weighted median | 10 | -0.375 | 0.257 | 1.44E-01 |
| Adiponectin | systolic blood pressure | Inverse variance weighted | 10 | -0.520 | 0.643 | 4.19E-01 |
| Adiponectin | systolic blood pressure | Simple mode | 10 | -0.454 | 0.374 | 2.55E-01 |
| Adiponectin | systolic blood pressure | Weighted mode | 10 | -0.372 | 0.248 | 1.68E-01 |
| High light scatter reticulocyte percentage | systolic blood pressure | MR Egger | 238 | 0.316 | 0.388 | 4.16E-01 |
| High light scatter reticulocyte percentage | systolic blood pressure | Weighted median | 238 | 0.271 | 0.167 | 1.03E-01 |
| High light scatter reticulocyte percentage | systolic blood pressure | Inverse variance weighted | 238 | 0.600 | 0.217 | 5.79E-03 |
| High light scatter reticulocyte percentage | systolic blood pressure | Simple mode | 238 | 0.082 | 0.359 | 8.19E-01 |
| High light scatter reticulocyte percentage | systolic blood pressure | Weighted mode | 238 | 0.286 | 0.131 | 3.03E-02 |
| Body fat percentage | systolic blood pressure | MR Egger | 221 | -5.047 | 1.833 | 6.38E-03 |
| Body fat percentage | systolic blood pressure | Weighted median | 221 | 0.460 | 0.352 | 1.92E-01 |
| Body fat percentage | systolic blood pressure | Inverse variance weighted | 221 | -0.647 | 0.511 | 2.06E-01 |
| Body fat percentage | systolic blood pressure | Simple mode | 221 | 1.364 | 0.945 | 1.50E-01 |
| Body fat percentage | systolic blood pressure | Weighted mode | 221 | 1.096 | 0.614 | 7.53E-02 |
| Vitamin D | systolic blood pressure | MR Egger | 51 | -0.003 | 0.365 | 9.93E-01 |
| Vitamin D | systolic blood pressure | Weighted median | 51 | -0.116 | 0.164 | 4.78E-01 |
| Vitamin D | systolic blood pressure | Inverse variance weighted | 51 | -0.243 | 0.277 | 3.82E-01 |
| Vitamin D | systolic blood pressure | Simple mode | 51 | -0.112 | 0.410 | 7.85E-01 |
| Vitamin D | systolic blood pressure | Weighted mode | 51 | -0.112 | 0.135 | 4.07E-01 |
| Nap during day | systolic blood pressure | MR Egger | 44 | -0.015 | 3.821 | 9.97E-01 |
| Nap during day | systolic blood pressure | Weighted median | 44 | 2.375 | 0.876 | 6.69E-03 |
| Nap during day | systolic blood pressure | Inverse variance weighted | 44 | 2.855 | 0.989 | 3.90E-03 |
| Nap during day | systolic blood pressure | Simple mode | 44 | 2.102 | 2.436 | 3.93E-01 |
| Nap during day | systolic blood pressure | Weighted mode | 44 | 2.190 | 1.557 | 1.67E-01 |
| Urea | systolic blood pressure | MR Egger | 118 | 0.737 | 1.226 | 5.49E-01 |
| Urea | systolic blood pressure | Weighted median | 118 | -0.075 | 0.340 | 8.25E-01 |
| Urea | systolic blood pressure | Inverse variance weighted | 118 | 0.831 | 0.543 | 1.26E-01 |
| Urea | systolic blood pressure | Simple mode | 118 | 1.882 | 0.853 | 2.93E-02 |
| Urea | systolic blood pressure | Weighted mode | 118 | -0.333 | 0.435 | 4.45E-01 |
| Neuroticism | systolic blood pressure | MR Egger | 10 | -39.564 | 14.373 | 2.50E-02 |
| Neuroticism | systolic blood pressure | Weighted median | 10 | 1.414 | 0.831 | 8.88E-02 |
| Neuroticism | systolic blood pressure | Inverse variance weighted | 10 | -0.160 | 2.335 | 9.45E-01 |
| Neuroticism | systolic blood pressure | Simple mode | 10 | 1.540 | 0.898 | 1.20E-01 |
| Neuroticism | systolic blood pressure | Weighted mode | 10 | 1.462 | 0.881 | 1.32E-01 |
| Glucose | systolic blood pressure | MR Egger | 76 | 0.312 | 0.663 | 6.40E-01 |
| Glucose | systolic blood pressure | Weighted median | 76 | 0.992 | 0.207 | 1.58E-06 |
| Glucose | systolic blood pressure | Inverse variance weighted | 76 | 0.655 | 0.404 | 1.05E-01 |
| Glucose | systolic blood pressure | Simple mode | 76 | 1.938 | 0.567 | 1.02E-03 |
| Glucose | systolic blood pressure | Weighted mode | 76 | 1.000 | 0.202 | 4.37E-06 |
| Immature reticulocyte fraction | systolic blood pressure | MR Egger | 166 | 0.608 | 0.503 | 2.29E-01 |
| Immature reticulocyte fraction | systolic blood pressure | Weighted median | 166 | 0.420 | 0.201 | 3.69E-02 |
| Immature reticulocyte fraction | systolic blood pressure | Inverse variance weighted | 166 | 0.519 | 0.287 | 7.02E-02 |
| Immature reticulocyte fraction | systolic blood pressure | Simple mode | 166 | 0.886 | 0.415 | 3.43E-02 |
| Immature reticulocyte fraction | systolic blood pressure | Weighted mode | 166 | 0.569 | 0.173 | 1.22E-03 |
| Alcoholic drinks per week | systolic blood pressure | MR Egger | 34 | 4.604 | 2.031 | 3.03E-02 |
| Alcoholic drinks per week | systolic blood pressure | Weighted median | 34 | 3.093 | 0.437 | 1.53E-12 |
| Alcoholic drinks per week | systolic blood pressure | Inverse variance weighted | 34 | 3.005 | 1.275 | 1.84E-02 |
| Alcoholic drinks per week | systolic blood pressure | Simple mode | 34 | 2.743 | 1.459 | 6.88E-02 |
| Alcoholic drinks per week | systolic blood pressure | Weighted mode | 34 | 3.001 | 0.440 | 8.83E-08 |
| Whole body fat mass | systolic blood pressure | MR Egger | 249 | -2.491 | 1.023 | 1.56E-02 |
| Whole body fat mass | systolic blood pressure | Weighted median | 249 | 0.075 | 0.242 | 7.57E-01 |
| Whole body fat mass | systolic blood pressure | Inverse variance weighted | 249 | -0.755 | 0.325 | 2.02E-02 |
| Whole body fat mass | systolic blood pressure | Simple mode | 249 | 0.631 | 0.613 | 3.04E-01 |
| Whole body fat mass | systolic blood pressure | Weighted mode | 249 | 0.631 | 0.417 | 1.31E-01 |
| body mass index | systolic blood pressure | MR Egger | 493 | -1.197 | 0.662 | 7.11E-02 |
| body mass index | systolic blood pressure | Weighted median | 493 | -0.054 | 0.205 | 7.92E-01 |
| body mass index | systolic blood pressure | Inverse variance weighted | 493 | -0.603 | 0.249 | 1.54E-02 |
| body mass index | systolic blood pressure | Simple mode | 493 | -0.275 | 0.816 | 7.37E-01 |
| body mass index | systolic blood pressure | Weighted mode | 493 | 0.668 | 0.402 | 9.73E-02 |
| Trunk fat-free mass | systolic blood pressure | MR Egger | 358 | -2.799 | 0.914 | 2.36E-03 |
| Trunk fat-free mass | systolic blood pressure | Weighted median | 358 | -1.995 | 0.258 | 1.12E-14 |
| Trunk fat-free mass | systolic blood pressure | Inverse variance weighted | 358 | -2.593 | 0.354 | 2.37E-13 |
| Trunk fat-free mass | systolic blood pressure | Simple mode | 358 | -1.582 | 1.411 | 2.63E-01 |
| Trunk fat-free mass | systolic blood pressure | Weighted mode | 358 | 0.241 | 2.625 | 9.27E-01 |
| Usual walking pace | systolic blood pressure | MR Egger | 26 | 40.484 | 8.838 | 1.21E-04 |
| Usual walking pace | systolic blood pressure | Weighted median | 26 | -2.407 | 1.209 | 4.64E-02 |
| Usual walking pace | systolic blood pressure | Inverse variance weighted | 26 | 0.546 | 2.722 | 8.41E-01 |
| Usual walking pace | systolic blood pressure | Simple mode | 26 | -2.928 | 2.003 | 1.56E-01 |
| Usual walking pace | systolic blood pressure | Weighted mode | 26 | -2.598 | 1.738 | 1.47E-01 |
| Peak expiratory flow (PEF) | systolic blood pressure | MR Egger | 73 | 1.763 | 2.996 | 5.58E-01 |
| Peak expiratory flow (PEF) | systolic blood pressure | Weighted median | 73 | -0.440 | 0.503 | 3.82E-01 |
| Peak expiratory flow (PEF) | systolic blood pressure | Inverse variance weighted | 73 | -1.132 | 0.798 | 1.56E-01 |
| Peak expiratory flow (PEF) | systolic blood pressure | Simple mode | 73 | 1.069 | 1.008 | 2.93E-01 |
| Peak expiratory flow (PEF) | systolic blood pressure | Weighted mode | 73 | 0.350 | 0.634 | 5.83E-01 |
| Sleep duration | systolic blood pressure | MR Egger | 37 | -0.853 | 5.313 | 8.73E-01 |
| Sleep duration | systolic blood pressure | Weighted median | 37 | -0.988 | 0.774 | 2.02E-01 |
| Sleep duration | systolic blood pressure | Inverse variance weighted | 37 | -0.765 | 1.352 | 5.71E-01 |
| Sleep duration | systolic blood pressure | Simple mode | 37 | -0.488 | 1.745 | 7.81E-01 |
| Sleep duration | systolic blood pressure | Weighted mode | 37 | -0.488 | 2.133 | 8.20E-01 |
| Mean corpuscular haemoglobin concentration | systolic blood pressure | MR Egger | 80 | -0.469 | 0.674 | 4.88E-01 |
| Mean corpuscular haemoglobin concentration | systolic blood pressure | Weighted median | 80 | -0.678 | 0.235 | 3.98E-03 |
| Mean corpuscular haemoglobin concentration | systolic blood pressure | Inverse variance weighted | 80 | -0.210 | 0.355 | 5.54E-01 |
| Mean corpuscular haemoglobin concentration | systolic blood pressure | Simple mode | 80 | -1.324 | 0.435 | 3.19E-03 |
| Mean corpuscular haemoglobin concentration | systolic blood pressure | Weighted mode | 80 | -0.938 | 0.276 | 1.08E-03 |
| Current tobacco smoking | systolic blood pressure | MR Egger | 15 | -4.159 | 11.842 | 7.31E-01 |
| Current tobacco smoking | systolic blood pressure | Weighted median | 15 | 1.051 | 1.664 | 5.28E-01 |
| Current tobacco smoking | systolic blood pressure | Inverse variance weighted | 15 | 1.200 | 3.051 | 6.94E-01 |
| Current tobacco smoking | systolic blood pressure | Simple mode | 15 | 1.822 | 2.705 | 5.12E-01 |
| Current tobacco smoking | systolic blood pressure | Weighted mode | 15 | 0.180 | 1.842 | 9.24E-01 |
| Calcium | systolic blood pressure | MR Egger | 160 | -0.086 | 0.503 | 8.64E-01 |
| Calcium | systolic blood pressure | Weighted median | 160 | 0.153 | 0.215 | 4.78E-01 |
| Calcium | systolic blood pressure | Inverse variance weighted | 160 | 0.757 | 0.281 | 7.08E-03 |
| Calcium | systolic blood pressure | Simple mode | 160 | 0.833 | 0.548 | 1.31E-01 |
| Calcium | systolic blood pressure | Weighted mode | 160 | -0.394 | 0.227 | 8.51E-02 |
| Neuroticism score | systolic blood pressure | MR Egger | 60 | -0.717 | 1.388 | 6.07E-01 |
| Neuroticism score | systolic blood pressure | Weighted median | 60 | 0.036 | 0.141 | 8.02E-01 |
| Neuroticism score | systolic blood pressure | Inverse variance weighted | 60 | 0.016 | 0.257 | 9.50E-01 |
| Neuroticism score | systolic blood pressure | Simple mode | 60 | 0.354 | 0.409 | 3.90E-01 |
| Neuroticism score | systolic blood pressure | Weighted mode | 60 | 0.413 | 0.310 | 1.88E-01 |
| Whole body water mass | systolic blood pressure | MR Egger | 366 | -3.173 | 0.876 | 3.35E-04 |
| Whole body water mass | systolic blood pressure | Weighted median | 366 | -2.159 | 0.265 | 3.54E-16 |
| Whole body water mass | systolic blood pressure | Inverse variance weighted | 366 | -2.639 | 0.343 | 1.46E-14 |
| Whole body water mass | systolic blood pressure | Simple mode | 366 | -2.642 | 1.263 | 3.71E-02 |
| Whole body water mass | systolic blood pressure | Weighted mode | 366 | -3.895 | 2.328 | 9.51E-02 |
| Trunk fat mass | systolic blood pressure | MR Egger | 248 | -2.576 | 1.082 | 1.80E-02 |
| Trunk fat mass | systolic blood pressure | Weighted median | 248 | 0.015 | 0.230 | 9.48E-01 |
| Trunk fat mass | systolic blood pressure | Inverse variance weighted | 248 | -1.159 | 0.337 | 5.84E-04 |
| Trunk fat mass | systolic blood pressure | Simple mode | 248 | 0.613 | 0.612 | 3.17E-01 |
| Trunk fat mass | systolic blood pressure | Weighted mode | 248 | 0.506 | 0.397 | 2.04E-01 |
| Sleeplessness / insomnia | systolic blood pressure | MR Egger | 25 | 2.394 | 8.896 | 7.90E-01 |
| Sleeplessness / insomnia | systolic blood pressure | Weighted median | 25 | -0.526 | 1.060 | 6.20E-01 |
| Sleeplessness / insomnia | systolic blood pressure | Inverse variance weighted | 25 | 2.954 | 2.933 | 3.14E-01 |
| Sleeplessness / insomnia | systolic blood pressure | Simple mode | 25 | 0.414 | 1.638 | 8.03E-01 |
| Sleeplessness / insomnia | systolic blood pressure | Weighted mode | 25 | -1.238 | 1.177 | 3.04E-01 |
| Aspartate aminotransferase | systolic blood pressure | MR Egger | 167 | -1.008 | 0.858 | 2.42E-01 |
| Aspartate aminotransferase | systolic blood pressure | Weighted median | 167 | 0.451 | 0.271 | 9.60E-02 |
| Aspartate aminotransferase | systolic blood pressure | Inverse variance weighted | 167 | 0.982 | 0.413 | 1.73E-02 |
| Aspartate aminotransferase | systolic blood pressure | Simple mode | 167 | 1.453 | 0.673 | 3.23E-02 |
| Aspartate aminotransferase | systolic blood pressure | Weighted mode | 167 | 0.865 | 0.290 | 3.26E-03 |
| Lipoprotein A | systolic blood pressure | MR Egger | 10 | 0.019 | 0.076 | 8.13E-01 |
| Lipoprotein A | systolic blood pressure | Weighted median | 10 | 0.058 | 0.040 | 1.50E-01 |
| Lipoprotein A | systolic blood pressure | Inverse variance weighted | 10 | 0.093 | 0.065 | 1.52E-01 |
| Lipoprotein A | systolic blood pressure | Simple mode | 10 | 0.285 | 0.137 | 6.74E-02 |
| Lipoprotein A | systolic blood pressure | Weighted mode | 10 | 0.084 | 0.039 | 5.96E-02 |
| Hip circumference | systolic blood pressure | MR Egger | 251 | -2.904 | 1.083 | 7.83E-03 |
| Hip circumference | systolic blood pressure | Weighted median | 251 | -1.027 | 0.235 | 1.29E-05 |
| Hip circumference | systolic blood pressure | Inverse variance weighted | 251 | -1.967 | 0.363 | 5.90E-08 |
| Hip circumference | systolic blood pressure | Simple mode | 251 | -0.778 | 0.827 | 3.48E-01 |
| Hip circumference | systolic blood pressure | Weighted mode | 251 | -0.044 | 0.422 | 9.16E-01 |
| Basophill percentage | systolic blood pressure | MR Egger | 85 | 0.363 | 0.603 | 5.49E-01 |
| Basophill percentage | systolic blood pressure | Weighted median | 85 | -0.414 | 0.267 | 1.20E-01 |
| Basophill percentage | systolic blood pressure | Inverse variance weighted | 85 | -0.420 | 0.297 | 1.57E-01 |
| Basophill percentage | systolic blood pressure | Simple mode | 85 | -0.395 | 0.537 | 4.64E-01 |
| Basophill percentage | systolic blood pressure | Weighted mode | 85 | -0.395 | 0.306 | 2.00E-01 |
| Alkaline phosphatase | systolic blood pressure | MR Egger | 230 | 0.159 | 0.201 | 4.28E-01 |
| Alkaline phosphatase | systolic blood pressure | Weighted median | 230 | 0.292 | 0.108 | 6.64E-03 |
| Alkaline phosphatase | systolic blood pressure | Inverse variance weighted | 230 | 0.284 | 0.146 | 5.26E-02 |
| Alkaline phosphatase | systolic blood pressure | Simple mode | 230 | 0.105 | 0.300 | 7.27E-01 |
| Alkaline phosphatase | systolic blood pressure | Weighted mode | 230 | 0.226 | 0.084 | 7.72E-03 |
| Total protein | systolic blood pressure | MR Egger | 172 | 0.355 | 0.731 | 6.28E-01 |
| Total protein | systolic blood pressure | Weighted median | 172 | 0.698 | 0.225 | 1.95E-03 |
| Total protein | systolic blood pressure | Inverse variance weighted | 172 | 1.355 | 0.326 | 3.31E-05 |
| Total protein | systolic blood pressure | Simple mode | 172 | 0.762 | 0.670 | 2.57E-01 |
| Total protein | systolic blood pressure | Weighted mode | 172 | 0.518 | 0.411 | 2.09E-01 |
| Red blood cell (erythrocyte) count | systolic blood pressure | MR Egger | 255 | -0.265 | 0.587 | 6.53E-01 |
| Red blood cell (erythrocyte) count | systolic blood pressure | Weighted median | 255 | 0.347 | 0.203 | 8.67E-02 |
| Red blood cell (erythrocyte) count | systolic blood pressure | Inverse variance weighted | 255 | 0.511 | 0.316 | 1.06E-01 |
| Red blood cell (erythrocyte) count | systolic blood pressure | Simple mode | 255 | 0.755 | 0.420 | 7.37E-02 |
| Red blood cell (erythrocyte) count | systolic blood pressure | Weighted mode | 255 | 0.445 | 0.171 | 9.74E-03 |
| Apoliprotein B | systolic blood pressure | MR Egger | 117 | 0.691 | 0.455 | 1.32E-01 |
| Apoliprotein B | systolic blood pressure | Weighted median | 117 | 0.436 | 0.164 | 7.66E-03 |
| Apoliprotein B | systolic blood pressure | Inverse variance weighted | 117 | 0.203 | 0.314 | 5.18E-01 |
| Apoliprotein B | systolic blood pressure | Simple mode | 117 | 0.849 | 0.349 | 1.66E-02 |
| Apoliprotein B | systolic blood pressure | Weighted mode | 117 | 0.522 | 0.132 | 1.33E-04 |
| Fasting glucose | systolic blood pressure | MR Egger | 30 | 1.982 | 1.805 | 2.82E-01 |
| Fasting glucose | systolic blood pressure | Weighted median | 30 | 2.195 | 0.393 | 2.41E-08 |
| Fasting glucose | systolic blood pressure | Inverse variance weighted | 30 | 1.302 | 0.845 | 1.23E-01 |
| Fasting glucose | systolic blood pressure | Simple mode | 30 | 2.147 | 0.748 | 7.55E-03 |
| Fasting glucose | systolic blood pressure | Weighted mode | 30 | 2.053 | 0.362 | 4.03E-06 |
| Trunk predicted mass | systolic blood pressure | MR Egger | 358 | -2.901 | 0.915 | 1.66E-03 |
| Trunk predicted mass | systolic blood pressure | Weighted median | 358 | -1.985 | 0.270 | 2.15E-13 |
| Trunk predicted mass | systolic blood pressure | Inverse variance weighted | 358 | -2.550 | 0.354 | 5.98E-13 |
| Trunk predicted mass | systolic blood pressure | Simple mode | 358 | -1.358 | 1.316 | 3.03E-01 |
| Trunk predicted mass | systolic blood pressure | Weighted mode | 358 | 0.179 | 2.545 | 9.44E-01 |
| Lymphocyte count | systolic blood pressure | MR Egger | 248 | 1.936 | 0.652 | 3.31E-03 |
| Lymphocyte count | systolic blood pressure | Weighted median | 248 | 0.289 | 0.182 | 1.13E-01 |
| Lymphocyte count | systolic blood pressure | Inverse variance weighted | 248 | 0.530 | 0.269 | 4.92E-02 |
| Lymphocyte count | systolic blood pressure | Simple mode | 248 | -0.207 | 0.409 | 6.14E-01 |
| Lymphocyte count | systolic blood pressure | Weighted mode | 248 | 0.209 | 0.269 | 4.39E-01 |
| Creatinine (enzymatic) in urine | systolic blood pressure | MR Egger | 21 | -3.605 | 7.398 | 6.32E-01 |
| Creatinine (enzymatic) in urine | systolic blood pressure | Weighted median | 21 | -3.276 | 0.986 | 8.86E-04 |
| Creatinine (enzymatic) in urine | systolic blood pressure | Inverse variance weighted | 21 | -1.847 | 1.719 | 2.83E-01 |
| Creatinine (enzymatic) in urine | systolic blood pressure | Simple mode | 21 | -4.082 | 2.320 | 9.38E-02 |
| Creatinine (enzymatic) in urine | systolic blood pressure | Weighted mode | 21 | -3.866 | 2.172 | 9.02E-02 |
| Platelet count | systolic blood pressure | MR Egger | 335 | 0.716 | 0.328 | 2.96E-02 |
| Platelet count | systolic blood pressure | Weighted median | 335 | 0.399 | 0.134 | 2.92E-03 |
| Platelet count | systolic blood pressure | Inverse variance weighted | 335 | 0.462 | 0.181 | 1.07E-02 |
| Platelet count | systolic blood pressure | Simple mode | 335 | 0.054 | 0.308 | 8.61E-01 |
| Platelet count | systolic blood pressure | Weighted mode | 335 | 0.141 | 0.127 | 2.67E-01 |

Supplementary Table 4. MR-egger intercept test results.

| exposure | outcome | egger_intercept | se | pval |
| --- | --- | --- | --- | --- |
| Trunk fat percentage | systolic blood pressure | 0.064 | 0.030 | 3.52E-02 |
| Total cholesterol | systolic blood pressure | -0.031 | 0.022 | 1.57E-01 |
| Gamma glutamyltransferase | systolic blood pressure | 0.034 | 0.013 | 1.08E-02 |
| Heel bone mineral density (BMD) T-score automated | systolic blood pressure | 0.022 | 0.015 | 1.44E-01 |
| Glycated haemoglobin | systolic blood pressure | 0.025 | 0.011 | 3.08E-02 |
| Whole body fat-free mass | systolic blood pressure | 0.015 | 0.014 | 2.85E-01 |
| Platelet distribution width | systolic blood pressure | 0.020 | 0.012 | 9.08E-02 |
| IGF-1 | systolic blood pressure | 0.041 | 0.012 | 4.72E-04 |
| White blood cell (leukocyte) count | systolic blood pressure | -0.007 | 0.016 | 6.34E-01 |
| HDL cholesterol | systolic blood pressure | -0.023 | 0.013 | 7.35E-02 |
| Triglycerides | systolic blood pressure | 0.044 | 0.013 | 1.03E-03 |
| Reticulocyte count | systolic blood pressure | 0.011 | 0.014 | 4.19E-01 |
| Waist-to-hip ratio | systolic blood pressure | 0.116 | 0.098 | 2.45E-01 |
| Apoliprotein A | systolic blood pressure | -0.014 | 0.014 | 3.11E-01 |
| Monocyte count | systolic blood pressure | 0.011 | 0.012 | 3.63E-01 |
| Fasting insulin | systolic blood pressure | 0.288 | 0.178 | 1.31E-01 |
| Weight | systolic blood pressure | 0.011 | 0.018 | 5.53E-01 |
| Birth weight of first child | systolic blood pressure | -0.074 | 0.133 | 5.82E-01 |
| Basal metabolic rate | systolic blood pressure | 0.013 | 0.014 | 3.59E-01 |
| Standing height | systolic blood pressure | -0.007 | 0.010 | 4.89E-01 |
| Total bilirubin | systolic blood pressure | 0.004 | 0.011 | 7.10E-01 |
| Drive faster than motorway speed limit | systolic blood pressure | -0.125 | 0.265 | 6.49E-01 |
| Overall health rating | systolic blood pressure | 0.177 | 0.094 | 6.65E-02 |
| Phosphate | systolic blood pressure | 0.013 | 0.019 | 4.89E-01 |
| Cigarettes per Day | systolic blood pressure | 0.008 | 0.030 | 8.04E-01 |
| Mean time to correctly identify matches | systolic blood pressure | -0.145 | 0.146 | 3.31E-01 |
| Mean corpuscular haemoglobin | systolic blood pressure | -0.010 | 0.010 | 2.73E-01 |
| Albumin | systolic blood pressure | 0.013 | 0.020 | 5.27E-01 |
| Urinary sodium-potassium ratio | systolic blood pressure | 0.201 | 0.115 | 9.52E-02 |
| Haematocrit percentage | systolic blood pressure | 0.004 | 0.018 | 8.45E-01 |
| Sitting height | systolic blood pressure | 0.003 | 0.014 | 8.22E-01 |
| Monocyte percentage | systolic blood pressure | -0.009 | 0.010 | 3.84E-01 |
| SHBG | systolic blood pressure | -0.048 | 0.012 | 1.25E-04 |
| Lymphocyte percentage | systolic blood pressure | 0.000 | 0.018 | 9.86E-01 |
| C-reactive protein | systolic blood pressure | 0.002 | 0.011 | 8.57E-01 |
| Cystatin C | systolic blood pressure | 0.011 | 0.012 | 3.48E-01 |
| Eosinophill percentage | systolic blood pressure | -0.025 | 0.015 | 8.10E-02 |
| Red blood cell (erythrocyte) distribution width | systolic blood pressure | 0.004 | 0.011 | 7.51E-01 |
| Neutrophill percentage | systolic blood pressure | 0.021 | 0.019 | 2.54E-01 |
| Getting up in morning | systolic blood pressure | -0.008 | 0.043 | 8.46E-01 |
| Platelet crit | systolic blood pressure | -0.038 | 0.012 | 1.80E-03 |
| Body fat | systolic blood pressure | -0.132 | 0.232 | 5.86E-01 |
| Morning/evening person (chronotype) | systolic blood pressure | 0.061 | 0.029 | 4.22E-02 |
| LDL direct | systolic blood pressure | -0.036 | 0.022 | 9.87E-02 |
| Direct bilirubin | systolic blood pressure | 0.000 | 0.016 | 9.83E-01 |
| Forced vital capacity (FVC) | systolic blood pressure | 0.009 | 0.022 | 6.71E-01 |
| Waist circumference | systolic blood pressure | 0.028 | 0.022 | 2.10E-01 |
| Neutrophill count | systolic blood pressure | 0.015 | 0.019 | 4.20E-01 |
| Mean corpuscular volume | systolic blood pressure | -0.024 | 0.010 | 1.47E-02 |
| Forced expiratory volume in 1-second (FEV1) | systolic blood pressure | 0.016 | 0.031 | 5.97E-01 |
| Cholesterol | systolic blood pressure | -0.024 | 0.020 | 2.23E-01 |
| Sodium in urine | systolic blood pressure | 0.104 | 0.128 | 4.22E-01 |
| Daytime dozing / sleeping (narcolepsy) | systolic blood pressure | -0.067 | 0.094 | 4.82E-01 |
| Pulse rate automated reading | systolic blood pressure | -0.001 | 0.022 | 9.77E-01 |
| Mean platelet (thrombocyte) volume | systolic blood pressure | -0.004 | 0.008 | 6.13E-01 |
| Testosterone | systolic blood pressure | -0.066 | 0.029 | 2.73E-02 |
| Mean sphered cell volume | systolic blood pressure | -0.024 | 0.011 | 2.41E-02 |
| Fluid intelligence score | systolic blood pressure | -0.163 | 0.087 | 6.98E-02 |
| Mean reticulocyte volume | systolic blood pressure | -0.029 | 0.011 | 8.89E-03 |
| Creatinine | systolic blood pressure | -0.011 | 0.019 | 5.47E-01 |
| High light scatter reticulocyte count | systolic blood pressure | 0.005 | 0.014 | 7.30E-01 |
| Alcohol intake frequency. | systolic blood pressure | 0.047 | 0.043 | 2.84E-01 |
| Impedance of whole body | systolic blood pressure | -0.031 | 0.018 | 9.69E-02 |
| Past tobacco smoking | systolic blood pressure | -0.094 | 0.102 | 3.59E-01 |
| Alanine aminotransferase | systolic blood pressure | 0.047 | 0.020 | 2.21E-02 |
| Heart rate | systolic blood pressure | 0.073 | 0.097 | 4.69E-01 |
| Reticulocyte percentage | systolic blood pressure | 0.012 | 0.014 | 4.12E-01 |
| telomere length | systolic blood pressure | -0.026 | 0.020 | 1.94E-01 |
| Urate | systolic blood pressure | 0.049 | 0.013 | 1.93E-04 |
| Birth weight | systolic blood pressure | -0.089 | 0.065 | 1.74E-01 |
| Haemoglobin concentration | systolic blood pressure | 0.008 | 0.017 | 6.22E-01 |
| type 2 diabetes | systolic blood pressure | 0.017 | 0.017 | 3.01E-01 |
| Adiponectin | systolic blood pressure | -0.013 | 0.072 | 8.61E-01 |
| High light scatter reticulocyte percentage | systolic blood pressure | 0.011 | 0.012 | 3.77E-01 |
| Body fat percentage | systolic blood pressure | 0.071 | 0.028 | 1.32E-02 |
| Vitamin D | systolic blood pressure | -0.018 | 0.018 | 3.17E-01 |
| Nap during day | systolic blood pressure | 0.033 | 0.043 | 4.41E-01 |
| Urea | systolic blood pressure | 0.003 | 0.030 | 9.32E-01 |
| Neuroticism | systolic blood pressure | 0.901 | 0.326 | 2.46E-02 |
| Glucose | systolic blood pressure | 0.014 | 0.021 | 5.14E-01 |
| Immature reticulocyte fraction | systolic blood pressure | -0.004 | 0.017 | 8.31E-01 |
| Alcoholic drinks per week | systolic blood pressure | -0.042 | 0.042 | 3.19E-01 |
| Whole body fat mass | systolic blood pressure | 0.037 | 0.021 | 7.49E-02 |
| body mass index | systolic blood pressure | 0.010 | 0.011 | 3.34E-01 |
| Trunk fat-free mass | systolic blood pressure | 0.003 | 0.014 | 8.07E-01 |
| Usual walking pace | systolic blood pressure | -0.427 | 0.092 | 1.03E-04 |
| Peak expiratory flow (PEF) | systolic blood pressure | -0.053 | 0.053 | 3.20E-01 |
| Sleep duration | systolic blood pressure | 0.001 | 0.070 | 9.86E-01 |
| Mean corpuscular haemoglobin concentration | systolic blood pressure | 0.010 | 0.021 | 6.51E-01 |
| Current tobacco smoking | systolic blood pressure | 0.054 | 0.115 | 6.47E-01 |
| Calcium | systolic blood pressure | 0.030 | 0.015 | 4.60E-02 |
| Neuroticism score | systolic blood pressure | 0.044 | 0.082 | 5.93E-01 |
| Whole body water mass | systolic blood pressure | 0.009 | 0.013 | 5.08E-01 |
| Trunk fat mass | systolic blood pressure | 0.031 | 0.022 | 1.69E-01 |
| Sleeplessness / insomnia | systolic blood pressure | 0.007 | 0.112 | 9.47E-01 |
| Aspartate aminotransferase | systolic blood pressure | 0.057 | 0.022 | 9.29E-03 |
| Lipoprotein A | systolic blood pressure | 0.066 | 0.041 | 1.47E-01 |
| Hip circumference | systolic blood pressure | 0.021 | 0.023 | 3.60E-01 |
| Basophill percentage | systolic blood pressure | -0.024 | 0.016 | 1.41E-01 |
| Alkaline phosphatase | systolic blood pressure | 0.007 | 0.008 | 3.66E-01 |
| Total protein | systolic blood pressure | 0.028 | 0.018 | 1.28E-01 |
| Red blood cell (erythrocyte) count | systolic blood pressure | 0.023 | 0.015 | 1.19E-01 |
| Apoliprotein B | systolic blood pressure | -0.027 | 0.018 | 1.43E-01 |
| Fasting glucose | systolic blood pressure | -0.021 | 0.049 | 6.72E-01 |
| Trunk predicted mass | systolic blood pressure | 0.006 | 0.014 | 6.78E-01 |
| Lymphocyte count | systolic blood pressure | -0.039 | 0.017 | 1.90E-02 |
| Creatinine (enzymatic) in urine | systolic blood pressure | 0.034 | 0.139 | 8.09E-01 |
| Platelet count | systolic blood pressure | -0.010 | 0.010 | 3.53E-01 |

Supplementary Table 5. Evidence of trait selection for the causal mediation analyses.

| trait | trait to t2d evidence | t2d to trait evidence | trait to sbp evidence | mediation analysis |
| --- | --- | --- | --- | --- |
| Fasting insulin | TRUE | FALSE | TRUE | TRUE |
| Trunk fat percentage | TRUE | FALSE | TRUE | TRUE |
| Hip circumference | TRUE | FALSE | TRUE | TRUE |
| Standing height | TRUE | FALSE | TRUE | TRUE |
| Aspartate aminotransferase | TRUE | FALSE | TRUE | TRUE |
| Heart rate | FALSE | FALSE | TRUE | FALSE |
| telomere length | FALSE | FALSE | TRUE | FALSE |
| Alcoholic drinks per week | FALSE | FALSE | TRUE | FALSE |
| Past tobacco smoking | FALSE | FALSE | TRUE | FALSE |
| Sitting height | FALSE | FALSE | TRUE | FALSE |
| Trunk fat-free mass | FALSE | FALSE | TRUE | FALSE |
| Trunk predicted mass | FALSE | FALSE | TRUE | FALSE |
| Birth weight of first child | FALSE | FALSE | TRUE | FALSE |
| Platelet count | FALSE | FALSE | TRUE | FALSE |
| Platelet crit | FALSE | FALSE | TRUE | FALSE |
| Total protein | FALSE | FALSE | TRUE | FALSE |
| Waist-to-hip ratio | TRUE | TRUE | TRUE | FALSE |
| body mass index | TRUE | TRUE | TRUE | FALSE |
| Birth weight | TRUE | TRUE | TRUE | FALSE |
| Weight | TRUE | TRUE | TRUE | FALSE |
| Whole body fat mass | TRUE | TRUE | TRUE | FALSE |
| Whole body fat-free mass | TRUE | TRUE | TRUE | FALSE |
| Whole body water mass | TRUE | TRUE | TRUE | FALSE |
| Basal metabolic rate | TRUE | TRUE | TRUE | FALSE |
| Impedance of whole body | TRUE | TRUE | TRUE | FALSE |
| Trunk fat mass | TRUE | TRUE | TRUE | FALSE |
| Forced vital capacity (FVC) | TRUE | TRUE | TRUE | FALSE |
| Forced expiratory volume in 1-second (FEV1) | TRUE | TRUE | TRUE | FALSE |
| Reticulocyte percentage | TRUE | TRUE | TRUE | FALSE |
| Reticulocyte count | TRUE | TRUE | TRUE | FALSE |
| High light scatter reticulocyte percentage | TRUE | TRUE | TRUE | FALSE |
| High light scatter reticulocyte count | TRUE | TRUE | TRUE | FALSE |
| Alanine aminotransferase | TRUE | TRUE | TRUE | FALSE |
| Gamma glutamyltransferase | TRUE | TRUE | TRUE | FALSE |
| Glycated haemoglobin | TRUE | TRUE | TRUE | FALSE |
| HDL cholesterol | TRUE | TRUE | TRUE | FALSE |
| SHBG | TRUE | TRUE | TRUE | FALSE |
| Triglycerides | TRUE | TRUE | TRUE | FALSE |
| Nap during day | FALSE | TRUE | TRUE | FALSE |
| Pulse rate automated reading | FALSE | TRUE | TRUE | FALSE |
| White blood cell (leukocyte) count | FALSE | TRUE | TRUE | FALSE |
| Platelet distribution width | FALSE | TRUE | TRUE | FALSE |
| Albumin | FALSE | TRUE | TRUE | FALSE |
| Calcium | FALSE | TRUE | TRUE | FALSE |
| Urate | FALSE | TRUE | TRUE | FALSE |
| Total cholesterol | TRUE | FALSE | FALSE | FALSE |
| Peak expiratory flow (PEF) | TRUE | FALSE | FALSE | FALSE |
| Apoliprotein B | TRUE | FALSE | FALSE | FALSE |
| IGF-1 | TRUE | FALSE | FALSE | FALSE |
| Adiponectin | FALSE | FALSE | FALSE | FALSE |
| Neuroticism | FALSE | FALSE | FALSE | FALSE |
| Body fat | FALSE | FALSE | FALSE | FALSE |
| Getting up in morning | FALSE | FALSE | FALSE | FALSE |
| Sleeplessness / insomnia | FALSE | FALSE | FALSE | FALSE |
| Current tobacco smoking | FALSE | FALSE | FALSE | FALSE |
| Fluid intelligence score | FALSE | FALSE | FALSE | FALSE |
| Mean time to correctly identify matches | FALSE | FALSE | FALSE | FALSE |
| Neuroticism score | FALSE | FALSE | FALSE | FALSE |
| Creatinine (enzymatic) in urine | FALSE | FALSE | FALSE | FALSE |
| Drive faster than motorway speed limit | FALSE | FALSE | FALSE | FALSE |
| Sleep duration | FALSE | FALSE | FALSE | FALSE |
| Red blood cell (erythrocyte) count | FALSE | FALSE | FALSE | FALSE |
| Haemoglobin concentration | FALSE | FALSE | FALSE | FALSE |
| Haematocrit percentage | FALSE | FALSE | FALSE | FALSE |
| Mean corpuscular haemoglobin concentration | FALSE | FALSE | FALSE | FALSE |
| Red blood cell (erythrocyte) distribution width | FALSE | FALSE | FALSE | FALSE |
| Mean platelet (thrombocyte) volume | FALSE | FALSE | FALSE | FALSE |
| Monocyte count | FALSE | FALSE | FALSE | FALSE |
| Lymphocyte percentage | FALSE | FALSE | FALSE | FALSE |
| Monocyte percentage | FALSE | FALSE | FALSE | FALSE |
| Neutrophill percentage | FALSE | FALSE | FALSE | FALSE |
| Eosinophill percentage | FALSE | FALSE | FALSE | FALSE |
| Alkaline phosphatase | FALSE | FALSE | FALSE | FALSE |
| Direct bilirubin | FALSE | FALSE | FALSE | FALSE |
| Creatinine | FALSE | FALSE | FALSE | FALSE |
| C-reactive protein | FALSE | FALSE | FALSE | FALSE |
| Cystatin C | FALSE | FALSE | FALSE | FALSE |
| Lipoprotein A | FALSE | FALSE | FALSE | FALSE |
| Phosphate | FALSE | FALSE | FALSE | FALSE |
| Total bilirubin | FALSE | FALSE | FALSE | FALSE |
| Fasting glucose | TRUE | TRUE | FALSE | FALSE |
| Overall health rating | TRUE | TRUE | FALSE | FALSE |
| Body fat percentage | TRUE | TRUE | FALSE | FALSE |
| Waist circumference | TRUE | TRUE | FALSE | FALSE |
| Heel bone mineral density (BMD) T-score automated | TRUE | TRUE | FALSE | FALSE |
| Usual walking pace | TRUE | TRUE | FALSE | FALSE |
| Lymphocyte count | TRUE | TRUE | FALSE | FALSE |
| Mean sphered cell volume | TRUE | TRUE | FALSE | FALSE |
| Immature reticulocyte fraction | TRUE | TRUE | FALSE | FALSE |
| Apoliprotein A | TRUE | TRUE | FALSE | FALSE |
| Cholesterol | TRUE | TRUE | FALSE | FALSE |
| Glucose | TRUE | TRUE | FALSE | FALSE |
| LDL direct | TRUE | TRUE | FALSE | FALSE |
| Cigarettes per Day | FALSE | TRUE | FALSE | FALSE |
| Urinary sodium-potassium ratio | FALSE | TRUE | FALSE | FALSE |
| Morning/evening person (chronotype) | FALSE | TRUE | FALSE | FALSE |
| Daytime dozing / sleeping (narcolepsy) | FALSE | TRUE | FALSE | FALSE |
| Alcohol intake frequency. | FALSE | TRUE | FALSE | FALSE |
| Sodium in urine | FALSE | TRUE | FALSE | FALSE |
| Mean corpuscular volume | FALSE | TRUE | FALSE | FALSE |
| Mean corpuscular haemoglobin | FALSE | TRUE | FALSE | FALSE |
| Neutrophill count | FALSE | TRUE | FALSE | FALSE |
| Basophill percentage | FALSE | TRUE | FALSE | FALSE |
| Mean reticulocyte volume | FALSE | TRUE | FALSE | FALSE |
| Urea | FALSE | TRUE | FALSE | FALSE |
| Testosterone | FALSE | TRUE | FALSE | FALSE |
| Vitamin D | FALSE | TRUE | FALSE | FALSE |

Supplementary Table 6. Multivariable MR analyses showing the direct effects of traits on SBP.

| exposure | mediator | outcome | effect | estimate | se | pval | condF |
| --- | --- | --- | --- | --- | --- | --- | --- |
| Fasting insulin | t2d | sbp | direct | 6.151 | 1.055 | 3.60E-08 | 9.2 |
| Trunk fat percentage | t2d | sbp | direct | -1.453 | 0.413 | 4.98E-04 | 24.6 |
| Hip circumference | t2d | sbp | direct | -2.310 | 0.344 | 8.10E-11 | 28.5 |
| Standing height | t2d | sbp | direct | -0.963 | 0.283 | 7.15E-04 | 43.0 |
| Aspartate aminotransferase | t2d | sbp | direct | 1.803 | 0.432 | 3.92E-05 | 27.0 |

Supplementary Table 7. Proportion mediated by type 2 diabetes in the causal mediation analyses.

| exposure | mediator | outcome | estimate indirect | se indirect | estimate direct | se direct | estimate total | se total | proportion_mediated (%) |
| --- | --- | --- | --- | --- | --- | --- | --- | --- | --- |
| Fasting insulin | t2d | sbp | 2.572 | 0.455 | 6.151 | 1.055 | 7.661 | 2.146 | 33.6 |
| Trunk fat percentage | t2d | sbp | 0.552 | 0.139 | -1.453 | 0.413 | -1.082 | 0.451 |  |
| Hip circumference | t2d | sbp | 0.341 | 0.134 | -2.310 | 0.344 | -1.967 | 0.363 |  |
| Standing height | t2d | sbp | -0.066 | 0.094 | -0.963 | 0.283 | -0.956 | 0.212 | 6.9 |
| Aspartate aminotransferase | t2d | sbp | 0.101 | 0.130 | 1.803 | 0.432 | 0.982 | 0.413 | 10.2 |
